# Differences and similarities in cortical bone of the femur between donors with and without type 2 diabetes

**DOI:** 10.1093/jbmr/zjaf173

**Published:** 2025-11-21

**Authors:** Emily Berestesky, Sasidhar Uppuganti, Daniel Y Dapaah, Daniel Fernandes, Nick Livingston, David Lutsky, Yumeng Zhang, Alicia M Hymel, Jacquelyn S Pennings, Paul Voziyan, Mark D Does, Thomas L Willett, Jeffry S Nyman

**Affiliations:** Department of Biomedical Engineering, Vanderbilt University, Nashville, TN 37232, United States; Department of Orthopaedic Surgery, Vanderbilt University Medical Center, Nashville, TN 37232, United States; Department of Orthopaedic Surgery, Vanderbilt University Medical Center, Nashville, TN 37232, United States; Composite Biomaterial Systems Lab, Department of Systems Design Engineering, University of Waterloo, Waterloo, ON N2L 3G1, Canada; Department of Medicine, Health and Society, Vanderbilt University, Nashville, TN 37232, United States; Department of Medicine, Health and Society, Vanderbilt University, Nashville, TN 37232, United States; Department of Medicine, Health and Society, Vanderbilt University, Nashville, TN 37232, United States; Department of Orthopaedic Surgery, Vanderbilt University Medical Center, Nashville, TN 37232, United States; Department of Medicine, Health and Society, Vanderbilt University, Nashville, TN 37232, United States; Department of Orthopaedic Surgery, Vanderbilt University Medical Center, Nashville, TN 37232, United States; Department of Orthopaedic Surgery, Vanderbilt University Medical Center, Nashville, TN 37232, United States; Department of Biostatistics, Vanderbilt University Medical Center, Nashville, TN 37203, United States; Department of Orthopaedic Surgery, Vanderbilt University Medical Center, Nashville, TN 37232, United States; Department of Biomedical Engineering, Vanderbilt University, Nashville, TN 37232, United States; Department of Electrical Engineering and Computer Science, Vanderbilt University, Nashville, TN 37212, United States; Composite Biomaterial Systems Lab, Department of Systems Design Engineering, University of Waterloo, Waterloo, ON N2L 3G1, Canada; Department of Biomedical Engineering, Vanderbilt University, Nashville, TN 37232, United States; Department of Orthopaedic Surgery, Vanderbilt University Medical Center, Nashville, TN 37232, United States; United States Department of Veterans Affairs, Tennessee Valley Healthcare System, Nashville, TN 37212, United States

**Keywords:** Advanced glycation end-products, strength, toughness, fracture toughness, thermal stability, microindentation, micro-computed tomography, dual-energy X-ray absorptiometry, fracture risk

## Abstract

For a given BMD, adults with type 2 diabetes (T2D) have greater fracture risk than adults without the disease. To test the hypothesis that T2D lowers the fracture resistance of human cortical bone by negatively altering the bone matrix quality, we acquired cadaveric femurs from 120 female and male donors >50 yr old: 60 without diabetes (Ctrl) and 60 with T2D for ≥10 yr. We scanned a cross-section from each diaphysis using ex vivo micro-CT (μCT), followed by cyclic reference point indentation (cRPI: 0-10 N for 20 cycles) and impact micro-indentation on the medial surface. From the medial quadrant, a tensile specimen and a single-edge notched beam (SENB) were mechanically tested to assess differences in fracture resistance. Multiple techniques characterized the organic matrix within the SENB. The cortical bone area and thickness of the diaphysis were higher in T2D than in Ctrl. The average creep indentation distance of periosteal bone tissue was significantly lower with T2D suggesting greater resistance to micro-indentation. Bone material strength index trended to be lowering in T2D than in Ctrl but only when the comparison was adjusted for age, sex, and BMI. There were also T2D-related differences in the organic matrix: (1) higher non-enzymatic and mature enzymatic crosslinks, (2) higher fluorescent advanced glycation end-products, and (3) higher thermal stability. Despite these tissue- and molecular-level differences, mechanical properties of cortical bone were similar between the 2 groups. Tensile strength was lower (*p* = .035), while pentosidine was higher (*p* = .006) in donors with CKD than donors without kidney disease, but the difference in strength (*p* = .055) and pentosidine (*p* = .151) were not strictly significant when adjusting for covariates. The elevated fracture risk in T2D may not be a problem of poor mechanical properties of cortical bone, despite alterations in the organic matrix.

## Introduction

Adults with type 2 diabetes (T2D; Table S1) are ~2 times more likely to experience a fragility fracture than age- and sex-matched adults without diabetes as determined by meta-analyses of fracture studies.^1^^,^^2^ With respect to problematic hip fractures, T2D increased fracture risk by ~20% in a retrospective study of women and men who were 66 yr or older.^3^ Likewise, in a longitudinal study of fragility fractures in women and in men, a 1-unit decrease in areal BMD (aBMD) of the femoral neck (FN) was associated with 1.88 higher risk of a hip fracture for women with T2D and associated with 5.71 higher risk of a hip fracture for men with T2D.^4^ Adults with T2D also had a higher fracture risk than adults without diabetes for a given T-score, age, or FRAX score.^4^ Thus, standard methods for predicting an individual’s fracture risk are not particularly effective when the patient has diabetes.

Although T2D is known to increase the risk of a fragility fracture for a given aBMD^2^ and standard osteoporosis treatments prevent fractures in adults with T2D,^5^^,^^6^ there is no therapeutic option specifically directed to inhibiting the pathogenic mechanism of bone fragility in T2D.^7^ Finding new solutions to prevent fragility fractures is critical, because: (1) fractures do not readily heal in the elderly and in those with T2D^8^ and (2) fractures reduce quality of life and increase the likelihood of death.^9^

Fracture risk in T2D is possibly a problem of bone *structural* quality and/or bone *matrix* quality. There have been cross-sectional and longitudinal studies comparing bone measurements by noninvasive or minimally invasive techniques between a T2D group and a nondiabetes (non-D) group with or without a fracture (Table S2). Cortical bone structure and trabecular architecture, as determined by HR-pQCT, either does not differ between groups or is better in T2D than in non-D.^10^^,^^11^ The exception is the comparison of fracture case vs no fracture case in patients with T2D. Then, the differences included higher cortical porosity in T2D subjects with a fracture.^12^^,^^13^ These clinical studies are equivocal on whether the elevated fracture in T2D is due to poor mechanical properties of bone at the apparent level (independent of bone size but not porosity and matrix composition) and/or poor organization of bone architecture.

Diabetes-related differences in bone can also be assessed using discarded bone samples acquired from orthopedic surgeries, namely total hip arthroplasty (THA) to treat end-stage osteoarthritis (OA) and hemi-arthroplasty (HA) to fix a fragility fracture of the FN. Regarding differences in mechanical properties of bone between T2D (*n* = 15) and non-D subjects (*n* = 23) without a fracture (~45% women), the apparent compressive stress (yield or ultimate) and toughness (total and post-yield) of trabecular bone was not affected by diabetes.^14^ Upon collecting proximal femurs from OA-THA cases involving postmenopausal women, apparent yield and ultimate stress in compression were also not different between T2D and non-D.^15^^,^^16^ However, apparent yield and ultimate compressive stress were significantly higher in T2D (*n* = 31 males) than in non-D (*n* = 34 males), with or without normalizing the mechanical property by trabecular bone volume fraction (BV/TV).^17^ Likewise, the T2D group (*n* = 17 males) had 39%-59% higher trabecular bone strength compared to the non-D group (*n* = 17 age-matched males).^18^ Thus, it is uncertain whether bone fragility in T2D is a problem of low strength and toughness of trabecular bone.

Another option to ascertain the origins of bone fragility in diabetes is to analyze cadaveric tissue from donors with and without T2D. Such an approach was used to identify the significant age-related changes in mechanical properties (strength, toughness, fatigue resistance, and fracture toughness), compositional property (collagen integrity), and microstructural (pores) properties of human cortical bone.^19^ To the best of our knowledge, there are only 2 studies comparing cadaveric bone between T2D and non-D donors.^20^^,^^21^ In the second study by the same research group involving the cadaveric femur mid-diaphysis from donors with T2D (11 elderly men; duration of disease not specified) and without T2D (12 elderly men), there were no significant differences in fracture toughness properties (transverse crack propagation) and nanoindentation properties (longitudinal surface).^21^

**Figure 1:**
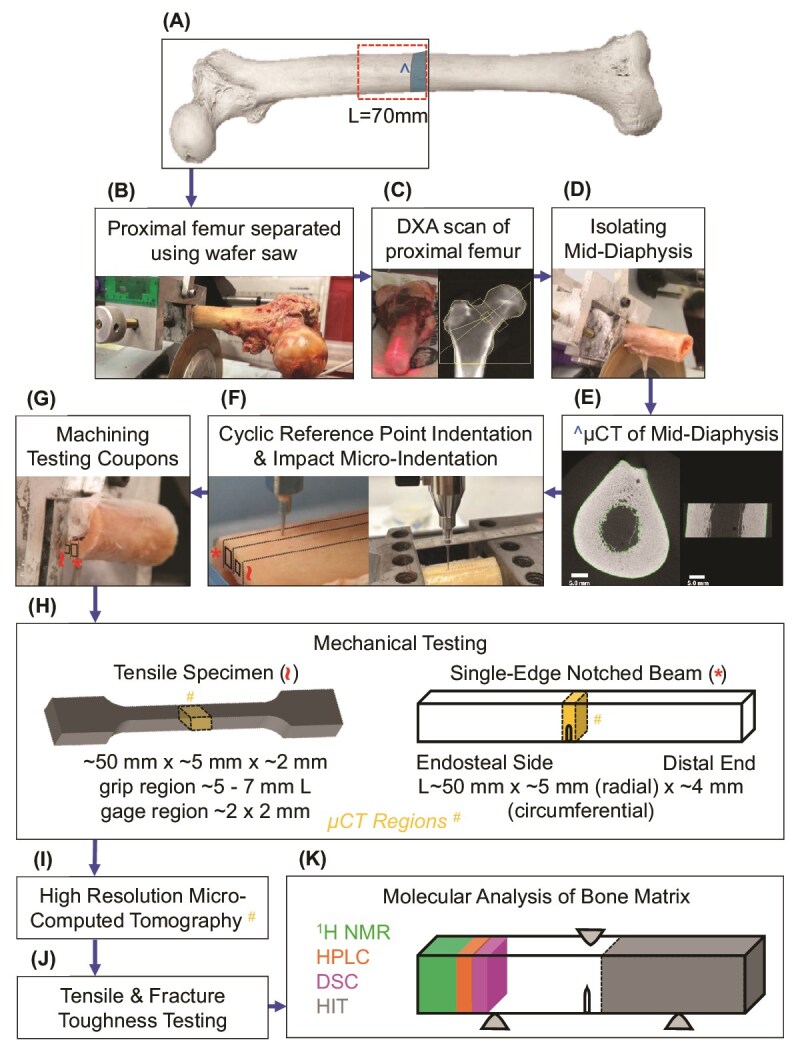
Overview of methods to assess bone properties of cadaveric femurs at different length scales. The midpoint of fresh-frozen, cadaveric femurs was measured and used to identify a 70 mm section of the proximal mid-diaphysis (A). The proximal femur was then separated from the distal portion at the midpoint using a low-speed diamond tipped wafer saw (B) and subjected to DXA scans (C). A cross-section with a length (*L*) of ~70 mm was isolated from the proximal femur (D) and scanned using micro-computed tomography (μCT) (E). Cyclic reference point indentation (cRPI) and impact micro-indentation (IMI) were done along parallel tracks on the medial surface of the mid-diaphysis using a BioDent and an OsteoProbe instrument, respectively (F). Following these indentation procedures, testing coupons were machined out of the medial quadrant (G) for tensile and fracture toughness testing (H). Specimens underwent high-resolution μCT to identify any sample variation prior to mechanical testing (I). Following tensile and fracture toughness testing (*J*), specimens were cut from SENB for hydrothermal isometric tension (HIT) testing, differential scanning calorimetry (DSC), high-performance liquid chromatography (HPLC) plus fluorescent AGEs, and proton NMR (^1^H NMR) relaxometry (K).

Because previous studies with limited sample size have not demonstrated that there are T2D-related deficits in the mechanical properties of human cortical bone, we collected 120 cadaveric femurs from 60 donors without diabetes and 60 donors with T2D for at least 10 yr, matching age and sex across the groups. The femurs were comprehensively analyzed (Figure 1) to test the hypothesis that T2D lowers the mechanical properties of human cortical bone by altering the tissue matrix. This hypothesis was initially based on the clinical observations that bone mass is higher, not lower, in T2D,^10^ thereby suggesting that a T2D-related deterioration in the inherent quality of bone tissue explains the higher fracture risk. In testing the hypothesis, we compared multiple bone characteristics between donors with T2D and control donors with and without adjusting for donor sex, age, and BMI, given that some bone characteristics can depend on these covariates. Assessment of matrix-related characteristics included glycation-mediated, non-enzymatic modifications as a potential explanation for T2D-related differences in mechanical properties, thereby supporting a treatment strategy of inhibiting these posttranslational modifications to prevent bone fragility. Lastly, in a sub-group analysis, we tested for significant differences in bone characteristics between donors with CKD and without CKD, again with and without including the same covariates, because CKD, a common complication of T2D, increases fracture risk.^22^

## Materials and methods

### Study design

One hundred and twenty, fresh-frozen, cadaveric femurs were obtained from the National Disease Research Interchange (NDRI) and Musculoskeletal Tissue Foundation. Sixty whole bones came from donors with T2D for a duration of 10 yr or greater, and 60 came from nondiabetic donors (Ctrl), as confirmed by information from the tissue banks who had access to limited medical records of the donors. The tissue banks indicated that the duration of T2D was “many years” for 3 donors, 2 of which had CKD and unknown for 4 donors, 2 of which had CKD. Diabetic donors were age-matched (±5 yr) to nondiabetic, control donors while maintaining sex equivalence (Table 1).

**Table 1:** Characteristics of the donors that provided cadaveric femurs.

**Characteristic**	**(Units) or category**	**Sample size**	**Ctrl** ^**a**^	**T2D** ^**a**^	***p*-value** ^**b**^
**Age**	(yr)	120	73 (64, 82)	72 (63, 82)	.893
**Age of female (F)**	(yr)	60	73 (60, 80)	71 (60, 80)	.857
**Age of male (M)**	(yr)	60	75 (65, 86)	75 (68, 85)	>.99
**Sex**	F/M	120	30/30 (50%)	30/30 (50%)	>.99
**BMI**	(kg/m^2^)	119^c^	26.6 (22.1, 32.9)	31.3 (26.7, 39.2)	**.002**
**BMI of female (F)** ^**h**^	(kg/m^2^)	59	25.4 (20.1, 32.4)	32.3 (25.9,43.2)	**.011**
**BMI of male (M)**	(kg/m^2^)	60	27.1 (23.9, 33.3)	30.1 (26.7, 36.9)	.068
**Duration of T2D**	(yr)	53^d^	N/A	20 (15, 27)	-
**Tissue bank**	NDRI femurs	120	43 (71.7%)	48 (80.0)	.286
**CKD**	No CKD	119^e^	53 (89.8%)	35 (58.3%)	**<.001**
	Stage 3	10	2	8	-
	Stage 4	3	0	3	-
	Stage 5	6	0	6	-
	Stage unknown	12	4	8	-
**Hypertension**	Total	91^f^	27 (62.8%)	40 (83.3%)	**.026**
**Cause of death** ^**g**^	Profile	119^h^	-	-	.090
	Brain	13	7 (11.7%)	6 (10.2%)	-
	Cardiovascular	66	29 (48.3%)	37 (62.7%)	-
	Respiratory	20	9 (15.0%)	11 (19.6%)	-
	Trauma	10	9 (15.0%)	1 (1.7%)	-
	Other	10	6 (10.0%)	4 (6.8%)	-

^a^Median (IQR); 100 × *n* of first category/total *n* (%).

^b^Wilcoxon–Mann–Whitney test or Pearson’s Chi-squared test.

^c^Missing information for 1 T2D donor who was a 62-yr-old female.

^d^Information was marked “unknown” or “many years” in the donor sheet.

^e^Missing information for 1 Control donor who was a 59-yr-old female.

^f^This information was only available from NDRI.

^g^Cardiovascular complication includes “Cardiac arrest,” “Heart failure,” and “Cardiopulmonary arrest,” respiratory failure includes “Respiratory arrest” and “Respiratory failure,” brain complication includes “Cerebral vascular accident” and “Stroke,” trauma includes “Trauma” and “Shock,” and other includes “Dementia,” “Natural causes,” “Other,” and “Renal failure.”

^h^Missing information for 1 T2D donor who was a 54-yr-old female. *p*-values <0.05 are bolded.

After measuring 70 mm proximal to the midpoint of the diaphysis (Figure 1A), a cross-section of the diaphysis was cut (Figure 1B) using a diamond-wheel saw (Model 660, South Bay Technology, Inc.). The proximal femur and mid-diaphysis of each cadaveric femur were evaluated for whole bone-level characteristics (Figure 1C). Following micro-CT (μCT) evaluations of mid-diaphysis (Figure 1D and E), impact micro-indentation (IMI) and cyclic reference point indentation (cRPI) was performed on the medial surface (Figure 1F). Next, 2 rectangular cortical strips with a thickness ~2-5 mm were extracted from the medial portion of the cross-section (Figure 1G) using the same saw. Each cortical strip was machined into a single-edge notched beam (SENB) and dog bone-shaped tensile coupon (Figure 1H) using an end-mill and constant irrigation for fracture toughness^23^ and tensile^24^ testing, respectively. This removed the indents. One face of each mechanical specimen was ground and polished as previously described.^23^^,^^25^ Test regions were scanned by μCT before mechanical testing (Figure 1I). After fracture toughness testing (Figure 1J), each SENB was sectioned (Figure 1K) for compositional analysis as described in subsequent sections.

### X-ray evaluations of proximal femur and mid-diaphysis

After thawing the bone to room temperature, each proximal femur was placed on top of a bag of rice (~4.5 kg) with the anterior side up so that the long axis of the femur could be aligned with the long axis of the scanner bed (Figure 1C). The rice attenuated X-ray intensity to mimic soft tissue attenuation. A DXA scan of the hip (length of 15.3 cm) was performed using a Horizon W (Hologic, Inc.): X-ray Mode 3, 140/100 kVp, 10 mA, and 31 s exposure time. A quality control phantom (Hologic aLumbar spine phantom) was scanned to verify that the scanner measured the reference values (BMD = 0.904 g/cm^2^; BMC = 49.499 g; Area = 54.768 cm^2^). Areal BMD was determined for the hip and FN using the manufacturer software.

After securing the cross-section of the femur mid-diaphysis in a tube (48 mm diameter) filled with PBS, a 13.16 mm segment near the mid-point was scanned using an ex vivo μCT scanner (μCT50, SCANCO Medical AG): X-ray source energy settings of 70 kVp and 200 μA, 0.5 mm Al filter between the source and bone, an integration time of 1.5 s, 1024 samples per projection, and 1000 projections per full rotation of the sample. The reconstructed images had an isotropic voxel size of 48.4 μm. Upon fitting a contour to the periosteal surface of each cross-sectional image, the Scanco script for mid-shaft evaluations was applied to each reconstructed image stack using a Gaussian image noise filter (sigma = 0.8, support = 1) and a global threshold for segmentation (bone voxels had a density between 613.7 and 2787.3 mg·HA/cm^3^). The script generated the following parameters: mean cross-sectional bone area (Ct.Ar), mean cross-sectional total area (Tt.Ar), mean cross-sectional moment of inertia (*I*_min_), mean distance between the centroid and outermost bone surface along minor axis (*c*_min_), and mean tissue mineral density (TMD), which is the hydroxyapatite (HA)-calibrated attenuation of all bone voxels (post segmentation) with a peel of 2 surface voxels.

To assess cortical porosity (Ct.Po) of the femur mid-diaphysis, another contiguous contour was drawn along the endosteum. This hand-drawn, circumferential contour was then tightly fitted to the endosteal surface (Figure S1) using the automated iterate forward function (inner and outer values were set to 200 and 500, respectively) of the contouring toolbox in the Scanco evaluation software. The morphed contours were checked across the entire image stack and manually fixed to avoid trabecular-like bone (Figure S1). With the ROI defined by the periosteal and endosteal contours, a global inverse threshold of <441.5 mgHA/cm^3^ (sigma = 0.2, support = 1) was used to determine Ct.Po as the pore voxel volume per total volume within the 2 contours and multiplied by 100 (Figure S1).

### Micro-indentation of mid-diaphysis

Each hydrated femur mid-shaft was secured in a vice for IMI using the OsteoProbe (ActiveLife Scientific, Inc.) and cRPI using the BioDent (ActiveLife Scientific, Inc.) along parallel tracks (Figure 1E). The OsteoProbe generated an impact force of ~40 N per indent, while the BioDent cyclically indented each site 20 times with a frequency of 2 Hz and a target force of 10 N.^26^ The spacing between the indents was 0.75-2 mm. After each series of indents by the OsteoProbe, a reference material of polymethylmethacrylate (PMMA) was indented 10 times with the same probe tip, which was replaced for each femur. A BP2 probe tip of the BioDent performed no more than 135 indents across different bones before being replaced with a new probe tip assembly. Similar reference blocks were indented (5 sites) before, in the middle, and at the end of the indents per BP2 probe tip. Cyclic reference point indentation properties of the PMMA did not vary among different BP2 probe tips, thereby ensuring consistent measurements throughout testing. Taking the harmonic mean of 10 indents on bone and 10 indents on PMMA, bone material strength index (BMSi) was the penetration depth of the tip in PMMA divided by the penetration depth for cortical bone. The 20 cycles of force vs displacement curves generated by the BioDent were analyzed as previously described^25^ to determine indentation distance increase (IDI), total indentation distance (TID), average energy dissipation (avg-ED) from third to last cycle, average creep indentation distance (avg-CID) from third to last cycle, and average loading and unloading slopes (avg-LS and avg-US) from third to last cycle (Figure S2).

### μCT analysis of mechanical specimens

As previously described,^23^^,^^24^ each test region of the mechanical specimens was scanned using the same Scanco μCT50 at higher resolution: 90 kVp and 200 μA, 0.5 mm Al filter, 0.4 s integration time, and 1024 samples for each of 1000 projections per full rotation. Placing the specimen in a tube (9 mm diameter) with PBS, the reconstructed voxel size was 5.0 μm. Fitting contours to the outer surface bone within each cross-section, volumetric BMD (vBMD) was the mean calibrated attenuation of all voxels and TMD was the calibrated attenuation of all bone voxels after segmentation (sigma = 1.8, support = 3, and threshold between 679.3 and 3000.0 mgHA/cm^3^). To determine the porosity of each specimen, we applied an inverse threshold to segment pore space from bone (sigma = 2, support = 2, and threshold between −500.0 and 616.4 mgHA/cm^3^) such that Ct.Po was the pore volume per total volume as a percentage.

### Mechanical testing of cortical bone

Each hydrated tensile specimen was loaded to failure at 5 mm/min using a material testing system (DynaMight 8841, Instron). With the specimen secured within grips (M10-82716-16, Instron), a dynamic extensometer (Model 2620-603, Instron) was attached to the gauge region. During *quasi*-static tension test, force from a 1000 N load cell (Dynacell, Instron) and displacement from the extensometer were recorded at 50 Hz. Prior to testing, the cross-sectional area of each specimen was measured using calipers. The mechanical properties were then calculated from the resulting engineering stress vs strain curve with the yield point being identified by the 0.2% offset method and toughness parameters being derived from the area-under-the-curve.^24^

To assess the ability of cortical bone to resist crack growth, each SENB was positioned horizontally on 2 supports with a span (S) equal to 4 times the width of the specimen. Using the same material testing system, the specimen was loaded in-line with the micro-notch such that the notch was subjected to Mode I opening. The force vs displacement data was recorded at 50 Hz as the hydrated bone was loaded in displacement control to failure with a progressive, multiple loading (+0.07 mm at 0.01 mm/s)–unloading (−0.04 mm at 0.015 mm/s)–dwell scheme. Since human cortical bone exhibits nonlinear mechanical behavior (ie, a significant amount of inelastic deformation at the notch tip), we calculated fracture toughness based on nonlinear elastic fracture mechanics.^23^

### Analysis of water compartments in bone samples

The volume fractions of bound water (BW) and pore water (PW) were quantified by ^1^H NMR relaxometry.^23^ Each hydrated bone sample from the SENB (Figure 1A) was sealed within a custom radiofrequency coil and inserted into a 4.7 T magnet (Varian). Using a Carr–Purcell–Meiboom–Gill pulse sequence (10 000 echoes at 100 μs echo spacing), ^1^H NMR signals were acquired and processed as previously described.^23^^,^^27^ By including a microsphere of water as a reference volume and measuring the volume of the bone specimen (Archimedes’ principle), the integrated areas of BW, PW, and microsphere of water components within the T_2_ spectrum were converted to volume of water (bound or pore) and then divided by volume of bone.

### Thermal stability of organic matrix

After cutting sections from each broken SENB specimen (Figure 1K), bone samples were demineralized in 20% ethylenediaminetetraacetic acid (EDTA) at room temperature for 5 wk. Once full demineralization was verified by X-ray imaging (Faxitron LX-60), samples were stored at −20 °C until further processing. Using a razor blade, beams were cut (~14 × ~2 mm × ~2 mm) from each de-mineralized cortical bone in preparation for hydrothermal isometric tension (HIT) testing. Cuboids (~3 mm × ~2 mm × ~1 mm) were also cut from the same de-mineralized bone for differential scanning calorimetry (DSC).

As previously described,^28^^,^^29^ the end of each demineralized bone was clamped such that the gauge length is fixed within a water bath (4 L) that is then heated from room temperature to 90 °C at ∼1.4 °C/min. Determined from the isometric stress (force/cross-sectional area of the sample) vs temperature curves, the temperature at which the organic matrix denatures (*T_d_*) and the maximum slope of change in stress per change temperature characterize thermal stability and connectivity of the organic matrix, respectively.^30^

After sealing demineralized bone sample in a DSC pan, the instrument (TA Instruments DSC Q-2000 System) measures the difference in heat flow between pan with the sample and an empty pan while heating both at a constant rate of 1.4 °C/min from 25 to 95 °C. The resulting endotherm characterizes the nativity and thermal stability of the bone collagen as follows: temperature at the onset of heat flow (*T*_onset_), the temperature at maximum recorded heat flow (*T*_peak_), and the enthalpy of denaturation (Δ*H*), which is the full-width at half maximum (FWHM) of the denaturation endotherm. The dry mass of each sample was obtained by freeze drying and re-weighing after DSC.

### Analysis of fluorescent advanced glycation end-products and collagen crosslinks

Sections (∼30 mg) from each SENB were demineralized in 20% EDTA at 4 °C for 5 wk and then hydrolyzed in 6 N HCl at 110 °C for 22 h. After hydrolysis, the hydrochloric acid was evaporated using a SpeedVac Concentrator System (Thermo Fisher Scientific) with a cold trap. Next, each hydrolysate was suspended in HPLC-grade water and centrifuged at 15 000 × *g* and 4 °C for 20 min. Aliquots corresponding to ~1 mg of dry bone were used for each assay.

Pentosidine (PEN), pyridinoline (PYD), and deoxypyridinoline (DPD) crosslinks were measured using a HPLC system (Agilent 1260 Infinity, Agilent Technologies) equipped with a Spherisorb 4.6 × 150 mm column (Waters Co.). The gradient program was 0.00-4.50 min, 87% mobile phase A (0.22% HFBA) and 13% mobile phase B (100% acetonitrile); 4.75-10.00 min, 83% A and 17% B; 10.25-16.00 min 75% A and 25% B; 16.75-21.00 min, 100% B; and 21.25-26.00 min, 13% B). The nonenzymatic and mature enzymatic crosslinks were calculated based on the standard curve and normalized to collagen levels measured in the same samples.^27^

Aliquots of hydrolyzed samples and quinine sulfate standards were dissolved in 0.1 M H_2_SO_4_. Briefly, 100 μL of sample and standards were pipetted in duplicate into a black-bottom 96-well plate. Fluorescence was measured at λex/λem = 370 nm/440 nm using BioTek Synergy H1 microplate reader (BioTek Instruments). Fluorescent advanced glycation end-products (fAGEs) levels were calculated based on the standard curve and normalized to collagen levels in the same samples.^28^

Hydroxyproline (Hyp) levels were measured by a colorimetric assay performed in 96-well plates following the addition of the oxidizer and Ehrlich’s solution to Hyp standards and the samples. The color was developed upon the incubation of the plates at 65 °C for 20 min and consequent rapid cooling. The measurements were taken at 550 nm using the same microplate reader and converted to mol of collagen, thereby normalizing fAGE and crosslink measurements.^27^

### Statistical analysis

Assuming diabetes is a type of accelerated aging, we estimated the sample size based on our previous study in which the correlation coefficient (*r*) between age and a fracture toughness property (ie, J-integral) was −0.38.^23^ To detect a difference between 2 groups for an effect size equal to 0.8216 (Cohen’s *d* based on *r*) using the Wilcoxon–Mann–Whitney test, a total sample size of 118 provides an α error probability equal to .05 and a power (1 − β probability error) equal to .99 (G^*^Power 3.1, Heinrich-Heine-Universität). Balancing the number of males (30 per group) and females (30 per group), a total sample size of 120 provides an α equal to .05 and a power equal to .959 in a general linear model without interaction terms that compares differences in properties between 2 groups (Ctrl and T2D) while adjusting for age, sex, and BMI.

Differences in bone properties and donor characteristics between Ctrl and T2D groups were tested for significance using the Wilcoxon–Mann–Whitney test. For categorical variables, including cause of death, Pearson’s Chi-squared test generated the *p*-value for the null hypothesis that the variable was equivalent across the 2 groups (R version 4.2). Each bone property was fit to linear regression models with group and sex as categorical variables and age and BMI as continuous variables (R version 4.2). For selected properties in which BMI was not significant (*p* > .1), we generated linear regression curves for plots of the property vs age based on estimated coefficients (GraphPad Prism version 10.4.1). The Wilcoxon–Mann–Whitney test and linear regression models were also used to determine if significant differences existed between donors without CKD and donors with CKD. Significant differences were reported when the *p*-value was less than .05, and trends were reported when the *p*-value was less than .1.

## Results

### BMI was higher in the T2D than in the Control (Ctrl) donors

While age- and sex-matched, the BMI was higher in the donors with T2D compared to Ctrl (Table 1). The duration of T2D varied between 10 and 40 yr with a median of 20 yr (Table 1). As might be expected, the incidence of donors with CKD or hypertension was higher in T2D than in Ctrl. The cause of death profile did not significantly vary between the 2 groups.

### Compared to Control (Ctrl) donors, femurs from T2D donors had higher bone mass, thicker cortices, and lower cortical porosity

**Figure 2:**
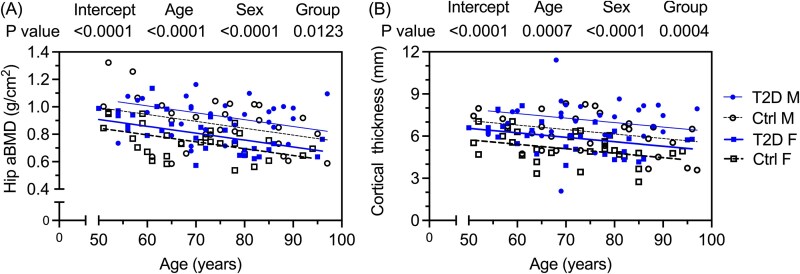
Linear regression of hip areal BMD (hip aBMD) of proximal femur and cortical thickness (Ct.Th) of the femur mid-diaphysis vs age. At a given age, both hip aBMD (A) and Ct.Th (B) are higher for donors who had T2D than for donors who didn’t have diabetes. *p*-values come from linear regression models in which group (Ctrl or T2D) and sex (male, M, or female, F) are categorical variables. For the estimates of coefficients for the full model that included BMI, which was not a significant explanatory variable, see Table S3.

The aBMD of the hip and FN was higher in T2D than in the Ctrl group (Table 2). When the comparison was adjusted for covariates, hip aBMD was still significantly higher in the diabetes group (Table S3). The apparent volumetric BMD (Ct.vBMD) and tissue mineral density (Ct.TMD) of the mid-diaphysis did not differ, but the cross-sectional cortical bone area (Ct.Ar) and the cortical thickness (Ct.Th) were significantly higher while cortical porosity (Ct.Po) was lower in T2D than in Ctrl with or without adjusting for covariates (Table 2 and Table S3). There were no diabetes-related differences in the resistance to bending factor known as section modulus (*I*_min_/*c*_min_) nor in the total cross-sectional area (Tt.Ar) (Table 2). Both hip aBMD and Ct.Th of the mid-diaphysis decreased with age such that each was higher in T2D than in Ctrl for a given age and within sex of the donor (Figure 2). Ct.Po increased with age and didn’t depend on sex or BMI of the donor such that cortical porosity of the mid-diaphysis was lower in T2D than in Ctrl for a given age (Table S3).

**Table 2:** Whole bone-level properties by DXA and μCT.

**Characteristic**	**(Units)**	**Ctrl** ^**a**^	**T2D** ^**a**^	***p*-value** ^**b**^	^*^ *p*-value ^**c**^
**Proximal femur (*n* = 57**-**60 per group)**^**d**^					
**Hip aBMD**	(g/cm^2^)	0.80 (0.67, 0.93)	0.88 (0.73, 0.99)	**.032**	**.046**
**Hip T-score**	-	−1.20 (−2.00, −0.10)	−0.55 (−1.70, 0.40)	**.041**	.070
**FN aBMD**	(g/cm^2^)	0.69 (0.59, 0.78)	0.76 (0.62, 0.83)	.061	.109
**FN T-score**	-	−1.40 (−2.30, −0.60)	−0.75 (−2.10, −0.13)	**.050**	.097
**Femur diaphysis (*n* = 60 per group)**					
***I***_**min**_ **/*c***_**min**_	(cm^3^)	1.53 (1.16, 1.92)	1.73 (1.35, 2.03)	.216	.216
**Ct.Ar**	(cm^2^)	3.73 (3.10, 4.56)	4.44 (3.57, 4.95)	**.012**	**.004**
**Tt.Ar**	(cm^2^)	6.01 (5.30, 6.97)	6.28 (5.37, 6.95)	.673	.629
**Ct.Th**	(mm)	5.56 (4.79, 6.64)	6.42 (5.80, 7.67)	**.002**	**.004**
**Ct.TMD**	(mg·HA/cm^3^)	862 (852, 868)	861 (848, 872)	.838	.669
**Ct.vBMD**	(mg·HA/cm^3^)	833 (807, 850)	844 (813, 862)	.115	.128
**Ct.Po**	(%)	6.66 (3.36, 11.2)	4.98 (2.58, 8.51)	**.026**	**.008**

^a^Median (IQR).

^b^Wilcoxon rank sum test (ie, Mann–Whitney U test).

^c*^ *p*-value for group (T2D or Ctrl) from linear regression models in which sex (male or female) and group were categorical variables while age (yr) and BMI (kg/cm^2^) were continuous variables. See Table S3 for the estimates of the coefficients and their respective *p*-values.

^d^Due to an implant in the proximal femur, aBMD could not be measured 1 male non-diabetic donor (96 yr) and 2 non-diabetic female donors (81 and 85 yr). *p*-values <0.05 are bolded.

### At the tissue-level, only the average of creep indentation distance was different between T2D and Ctrl

Average creep indentation distance (avg-CID) was significantly lower in the T2D donors compared to control donors (Figure S3), while there were no significant differences in TID and the other cRPI-derived mechanical properties between donor groups (Table 3). The clinical measurement of the resistance of cortical bone tissue to IMI, BMSi, was also not significantly different between groups (Table 3). Based on linear regression analysis (Table S3), Ctrl donors tended to have higher BMSi than T2D donors (*p* = .0962) for a given age or BMI in which BMSi significantly depended on the sex (lower in female) and age (gradual increase) of the donor (Figure S4).

**Table 3:** Mechanical properties of tissue by cyclic reference point indentation (cRPI), impact micro-indentation (IMI), tensile testing, and fracture toughness testing.

**Characteristic**	**(Units)**	**Ctrl** ^**a**^	**T2D** ^**a**^	***p*-value** ^**b**^	^*^ *p*-value ^**c**^
**BioDent cRPI from femoral periosteal surface (*n* = 60 per group)**					
**TID**	(mm)	81 (78, 87)	82 (78, 86)	.783	.318
**IDI**	(mm)	12.01 (11.42, 13.25)	11.76 (10.62, 13.14)	.187	.309
**avg-CID**	(mm)	1.46 (1.33, 1.61)	1.36 (1.27, 1.50)	**.019**	**.005**
**avg-ED**	(mJ)	47 (42, 53)	47 (41, 52)	.692	.579
**avg-US**	(N/mm)	0.42 (0.38, 0.44)	0.42 (0.39, 0.44)	.715	.536
**avg-LS**	(N/mm)	0.57 (0.51, 0.60)	0.57 (0.52, 0.60)	.669	.226
**OsteoProbe IMI (*n* = 60 per group)**					
**BMSi**	-	90 (86, 94)	89 (84, 92)	.231	.096
**Tensile specimens from femoral cortex (*n* = 60 per group)**					
**Ct.Po**	(%)	4.5 (3.1, 8.9)	4.3 (2.7, 6.1)	.236	.279
**Ct.TMD**	(mgHA/cm^3^)	1018 (977, 1039)	1022 (1000, 1049)	.209	.250
**Ct.vBMD**	(mgHA/cm^3^)	1069 (1055, 1079)	1067 (1054, 1080)	.747	.592
**Yield Stress**	(MPa)	96 (92, 103)	97 (89, 104)	.964	.943
**Ultimate Stress**	(MPa)	109 (100113)	106 (97, 116)	.503	.398
**Failure Stress**	(MPa)	109 (100, 112)	106 (97, 116)	.524	.443
**Yield Strain**	(%)	0.78 (0.76, 0.79)	0.77 (0.75, 0.78)	.082	.361
**Failure Strain**	(%)	2.0 (1.6, 2.2)	1.9 (1.6, 2.5)	.910	.302
**PY Strain**	(%)	1.2 (0.8, 1.4)	1.1 (0.8, 1.7)	.759	.513
**Toughness**	(MJ/m^3^)	1.67 (1.17, 2.00)	1.64 (1.20, 2.27)	.985	.618
**PY toughness**	(MJ/m^3^)	1.23 (0.74, 1.51)	1.15 (0.76, 1.80)	.811	.489
**Fracture toughness specimens from femoral cortex (*n* = 60 per group)**					
**Ct.Po**	(%)	4.2 (2.8, 6.9)	3.7 (2.6, 4.8)	**.050**	**.040**
**Ct.TMD**	(mgHA/cm^3^)	996 (984, 1007)	998 (988, 1010)	.391	.197
**Ct.vBMD**	(mgHA/cm^3^)	954 (925, 971)	966 (944, 983)	**.030**	**.025**
**K**_**init**_	MPa·(m)^0.5^	7.31 (6.28, 8.14)	6.77 (5.84, 8.14)	.372	.663
**J**_**f**_	kJ/m^2^	7.90 (6.68, 10.58)	7.92 (6.66, 9.88)	.751	.553
**K**_**grow**_	MPa·(m)^0.5^/(mm)^0.5^	5.30 (4.44, 6.76)	6.58 (4.89, 7.87)	.094	.700

^a^Median (IQR).

^b^Wilcoxon rank sum test (ie, Mann-Whitney U test).

^c*^ *p*-value for group (T2D or Ctrl) from linear regression models in which sex (male or female) and group were categorical variables while age (yr) and BMI (kg/cm^2^) were continuous variables. See Table S3 for the estimates of the coefficients and their respective *p*-values. *p*-values <0.05 are bolded.

### There were no significant differences in the mechanical properties of cortical bone between Ctrl and T2D

Checked by high-resolution μCT, the cortical porosity, Ct.vBMD, and Ct.TMD in the tensile specimens were not significantly different between groups, while Ct.Po and Ct.vBMD in the SENB specimens were lower and higher for the donors with T2D, respectively (Table 3). For tensile testing, ultimate strength, overall toughness, and other mechanical properties did not significantly differ between T2D and Ctrl donors (Table 3). For fracture toughness testing, critical stress intensity for crack initiation (*K*_init_), energy dissipated during crack growth until failure (*J_f_*), and the rate of crack propagation (*K*_grow_) did not depend on diabetic status of the donor. Ct.Po and Ct.vBMD can influence fracture toughness properties, but the ability of either Ct.Po (significant negative predictor) or Ct.vBMD (non-significant positive predictor) to explain the variance in fracture toughness properties did not depend on diabetic status when accounting for age and sex (Table S4).

### For a given age, advanced glycation end-products and the thermal stability of the organic matrix were higher in T2D than in Ctrl donors

**Figure 3:**
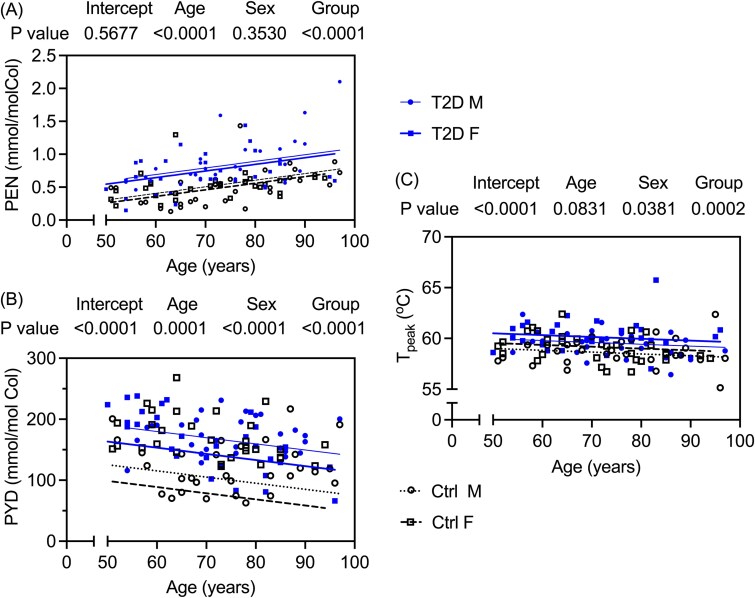
Linear regression of Pentosidine (PEN), Pyridinoline (PYD), and thermal stability (*T*_peak_) of demineralized cortical bone vs age. At a given age, both (A) Pentosidine (PEN) and (B) Pyridinoline (PYD) levels were elevated in the cortical bone of T2D donors compared to donors without diabetes. Additionally, the (C) peak temperature (*T*_peak_) during denaturation of the organic matrix, a measure of thermal stability by DSC, was greater for donors with T2D than for donors without diabetes. *p*-values come from general linear regressions in which group (Ctrl or T2D) and sex (male, M, or female, F) are categorical variables. For the estimates of coefficients for the full model that includes BMI, which was not a significant explanatory variable, see Table S3.

There was no significant difference in maximum slope, a measure of collagen network connectivity, during HIT testing between donor groups. The organic matrix from T2D donors however experienced denaturation at higher temperatures (*T_d_*) compared to controls (Table 4), even after adjustment for covariates (Table S3). Additionally, other measurements of the thermal stability of demineralized bone were significantly elevated in T2D than in Ctrl (Table 4). HPLC revealed a greater concentration of PEN, a nonenzymatic, glycation-mediated collagen crosslink, and greater amount of fAGEs in the bone of T2D donors compared to Ctrl donors (Table 4 and Table S3). Two mature enzymatic collagen crosslinks called PYD and DPD were also higher with T2D (Table 4). Based on linear regression models without BMI as a covariate (ie, BMI was not significant when included, Table S3), PEN levels (Figure 3A) and PYD (Figure 3B) levels significantly increased and decreased with age, respectively. For a given age and sex, thermal stability of demineralized bone was higher in T2D than in Ctrl (Figure 3C). There were no differences in BW and PW levels between groups (Table 4).

**Table 4:** Bone composition by hydrothermal isometric tension (HIT), differential scanning calorimetry (DSC), HPLC, micro-CT (μCT), and NMR relaxometry of specimens from SENB.

**Characteristic**	**(Units)**	**Ctrl** ^**a**^	**T2D** ^**a**^	***p*-value** ^**b**^	^^*^^ *p*-value
**HIT (*n* = 60 per group)**					
**Max slope**	(kPa)	45.8 (40.9, 52.8)	46.8 (41.5, 53.4)	.616	.601
***T***_***d***_	(°C)	62.9 (62.2, 63.7)	63.4 (62.9,64.0)	**.006**	**.005**
**DSC (*n* = 60 per group)**					
***T***_**onset**_	(°C)	52.0 (51.1, 53.3)	53.4 (51.9, 54.6)	**.002**	**.014**
***T***_**peak**_	(°C)	58.7 (58.0, 59.7)	59.8 (58.8, 60.6)	**<.001**	**.001**
**Δ*H***	(°C)	10.06 (8.82, 10.83)	9.34 (8.12, 10.62)	.097	.531
**HPLC (*n* = 60 per group)**					
**PEN**	(mmol/mol col)	0.518 (0.320, 0.615)	0.718 (0.598, 0.925)	**<.001**	**<.0001**
**PYD**	(mmol/mol col)	145 (109, 167)	171 (143, 199)	**.003**	**.010**
**DPD**	(mmol/mol col)	92 (66, 122)	111 (92, 129)	**.011**	**.029**
**fAGE**	(ng quinine/mol col)	43 (37, 50)	50 (42, 57)	**.002**	**.002**
^**1**^ **H NMR (*n* = 60 per group)**					
**Bound water**	(%)	20.2 (19.3, 20.7)	20.2 (19.6, 20.8)	.505	.449
**Pore water**	(%)	5.4 (4.3, 7.1)	5.1 (4.5, 6.3)	.243	.226

^a^Median (IQR).

^b^Wilcoxon rank sum test (ie, Mann–Whitney U test).

^c*^ *p*-value for group (T2D or Ctrl) from linear regression models in which sex (male or female) and group were categorical variables while age (yr) and BMI (kg/cm^2^) were continuous variables. See Table S3 for the estimates of the coefficients and their respective *p*-values. *p*-values <0.05 are bolded.

### Ultimate tensile stress was lower while pentosidine level was higher in donors with CKD than in donors without kidney disease

**Figure 4:**
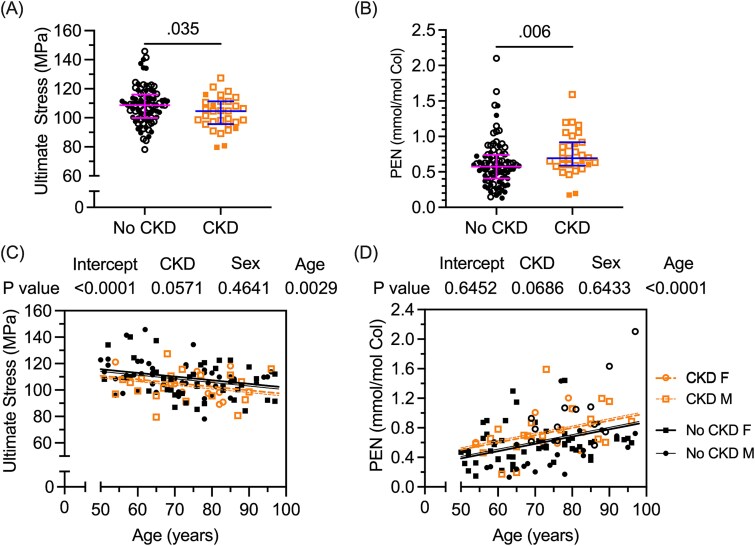
Differences in tensile strength and pentosidine level of cortical bone between donors with and without CKD. The ultimate stress was lower in CKD than in no CKD donors (A), while pentosidine level (PEN) was higher in the donors with CKD (B). Open symbols denote T2D donors; closed symbols denote Ctrl donors. When accounting for age-related changes in these bone properties, ultimate stress was lower in CKD than in no CKD for a given age, though confidence in this CKD effect was reduced (C). Likewise, PEN was higher in CKD than in no CKD for a given age, but the confidence in the CKD effect was also reduced (D).

Since CKD increases fracture risk and T2D increases likelihood of developing this condition, we performed a secondary analysis comparing the bone properties between donors with and without CKD. Information about kidney problems was insufficient for one donor, so there were 88 donors without CKD and 31 donors with apparent CKD, 4 of whom did not have diabetes (Table 1). Without adjusting for covariates, the only 2 bone properties to significantly vary (*p* < .05) between no CKD and CKD (Table S5) were ultimate tensile stress (Figure 4A) and PEN concentration (Figure 4B) of cortical bone. The level of confidence in a significant difference decreased when the comparisons were adjusted for covariates (Table S5). For a given age, ultimate tensile stress and PEN trended to being lower (Figure 4C) and higher (Figure 4D), respectively, in the CKD group compared to the no CKD group. The trend in a lower *K*_init_ with CKD (*p* = .068) was clearly not significant after adjustments (*p* = .416). Interestingly, there were no significant differences in hip aBMD and FN aBMD unless the comparison was adjusted for covariates (Table S5). For a given age or BMI, both DXA measurements were lower in CKD than in no CKD (Table S6).

## Discussion

Disproving our hypothesis, the apparent-level mechanical properties of human cortical bone were not different between donors with T2D and donors without diabetes, even though T2D altered tissue-level properties. This suggests that the elevated fracture risk in T2D—despite higher bone mass—cannot be attributed to an accelerated deterioration in the matrix causing a reduction in strength, toughness, and fracture toughness of cortical bone. Tensile strength (Figure 4A) was lower in donors with CKD compared to donors without CKD, though the confidence in this difference being significant was less when accounting for sex and age (Figure 4C). The present study did not specifically choose a sample size to detect CKD-related differences in bone properties, but the opportunistic number of donors with and without CKD (Table 1) meant that we were powered at 80% (α = .05) to detect differences greater than a moderate effect size of *d* = 0.60.

In the epidemiological studies that estimated the relative risk of a fragility facture among patients with T2D, a few investigated whether the higher fracture risk was significantly associated with T2D-related complications.^31^ In addition to an increase in fracture risk, as the duration of diabetes increases^32^ and glycemic control decreases,^33^ microvascular complications, stroke, cardiovascular disease, and kidney disease have been associated with higher relative risk of a fracture in T2D.^34^^,^^35^ Diabetic retinopathy and diabetic neuropathy are also T2D-related complications associated with elevated fracture risk as they increase the likelihood of falling.^35^^,^^36^ In a comparison of bone characteristics between T2D patients who sustained a hip fracture and healthy control donors, BV/TV was significantly lower only when T2D accompanied microvascular disease.^37^^,^^38^ Like clinical studies of T2D complications involving HR-pQCT,^39^ the present study supports the need for more studies investigating relationships between fracture risk and T2D-related comorbidities with respect to glycemic control.

Since AGEs result from chemical reactions between sugars and proteins, AGEs would be expected to be higher in T2D than in non-D bone. Oren et al. observed this for PEN but did not find significant differences in the enzymatic, PYD collagen crosslinks.^40^ This increase in an AGE was observed in subsequent studies comparing bone characteristics of discarded proximal femurs between non-D and T2D.^14^^,^^17^^,^^41^ In the present study of cortical bone with a large sample size, nonenzymatic PEN, enzymatic PYD, enzymatic DPD, and overall fAGEs were all significantly higher in T2D than in non-D (Ctrl) without and with adjustments for age, sex, and BMI of the donor (Table 4). This is possibly due to the low bone turnover that has been observed in T2D subjects when compared to subjects without diabetes.^42^ With low bone turnover, there is more time for glucose (ie, Maillard reaction) to form AGEs.

Since there were no differences in bone strength, toughness, and fracture toughness for a cohort of donors in which fracture history was unknown, the present study does not support the postulated pathogenic mechanism, whereby the accumulation of AGEs in T2D causes the bone to become brittle such that the ability of collagen fibrils to dissipate energy is impeded. Our findings agree with other studies involving bone samples from patients. Hunt et al. found higher PEN in patients with T2D but no significant diabetes-related differences in post-yield strain, post-yield toughness, and overall toughness of trabecular bone in compression.^17^ In another study, the levels of carboxymethyllysine were also significantly higher (*p* ≤ .001) in T2D than in non-D, regardless of whether trabecular or cortical bone was analyzed, but toughness (post-yield and total, *p* < .018) was higher, not lower, in T2D than in non-D group.^18^ There were no significant differences in fatigue resistance of trabecular bone and in nanomechanical properties of tissue (trabecular and cortical).^18^ In other studies using trabecular and cortical bone samples from fracture patients, fAGE levels were significantly higher (*p* = .015), while the mechanical properties in compression (eg, ultimate stress and toughness) were lower in the T2D group compared to non-D group.^41^^,^^43^ These studies did not include non-fracture cases, so it is unclear if the fracture resistance of bone is lower in T2D than in non-diabetes preceding a fragility fracture.

The present study found a complex relationship between BMSi and T2D that is partially concordant with other OsteoProbe studies. In a previous study involving the mid-diaphysis of cadaveric tibia from donors with T2D (6 women and 5 men; duration of disease not specified) and without T2D (3 women and 12 men), there was also no significant difference in BMSi between the 2 groups.^20^ Clinical studies involving men only^44^ or men and women^45^ also did not detect significant differences in BMSi. Upon adjusting the comparison between groups for sex, age, and BMI, BMSi of the cadaveric femur trended toward being lower in the T2D than the Ctrl group (Figure 3; Table S3). Bone material strength index was also lower in female donors than in male donors by 5.7 units (Figure 3; Table S3). This observation fits normative data (197 males and 282 females, ages 25-98 yr) acquired from OsteoProbe measurements.^46^ The age-related increase in BMSi however does not match clinical observation,^46^ but this increase was gradual in the present study (Figure 3). Likewise, BMSi probably does not increase with BMI as several clinical OsteoProbe studies found a weak, negative association between the 2 measurements.^44^^,^^47^^,^^48^ As an indicator of bone fragility in T2D, BMSi likely needs to be assessed in clinical studies of fracture risk while adjusting for at least sex and BMI.

The BioDent indenter was previously applied to the periosteal surface of the anterior-medial quadrant (cadaveric tibia mid-diaphysis) following fixation and storage in 70% ethanol (10 cycles of indentation with maximum force of 6 N).^20^ When 5 of 15 T2D donors were grouped based on high porosity (extensive endocortical “trabecularization”), only TID was higher and average loading slope (avg-LS) was lower compared to the non-D (*n* = 15) and T2D donors with normal porosity (*n* = 10). In the present study of 120 donors and cRPI on the periosteal, medial surface of non-fixed, hydrated tissue (femur mid-diaphysis), avg-CID was the only resistance to cyclic indentation property that differed between T2D and non-D groups (Table 3 and Table S3). Since avg-CID was lower in the T2D group than in the Ctrl group, the diabetic bone tissue was less resistant to creep under a constant force. In our previous study comparing different cRPI properties to mechanical properties from three-point bend testing of 34 uniform cortical beams (22 males and 12 females), the IDI had the strongest correlation with ultimate stress and IDI directly correlated with avg-CID.^25^ Thus, a lower avg-CID would suggest stronger bone and less likely to fracture.

In a recent cadaver study, Emerzian et al. analyzed cortical bone from female and male donors with type 1 diabetes for 50 yr or more and age-, sex-, and BMI-matched donors without diabetes.^49^ There were no significant differences in total hip areal BMD (DXA of proximal femur), BMSi (OsteoProbe of medial mid-diaphysis), all cRPI properties (BioDent of cortical beam), cortical TMD and porosity (μCT of cortical beam), and AGEs, including CML. Post-yield toughness and toughness (four-point bending of cortical beams) however were significantly lower in the T1D group than in control group, while PEN level was higher with T1D.^49^ Possibly, when T1D is managed for over 50 yr, the accumulation of a non-enzymatic, glycation-mediated collagen crosslink is sufficient to cause a reduction in the toughness of cortical bone.

In another study of cadaveric femurs comparing donors without (*n* = 18; 61-89 yr) and with T2D (*n* = 11; 69-89 yr), there were no significant differences in cortical porosity and TMD of SENB (anterior-lateral quadrant) as determined by ex vivo μCT.^21^ In our fracture toughness specimens, Ct.TMD was not different (Table 3), but Ct.Po of SENB was significantly but marginally lower in T2D than in Ctrl (Tables S3 and S4). Ct.Po was a negative predictor of fracture toughness, but being a T2D donor did not change how Ct.Po related to fracture toughness (Table S4). Regardless, both studies did not find a difference in the fracture toughness properties. In their analysis of cadaveric femurs from 43 male and 37 female donors, Dapaah et al. also did not find a difference in fracture toughness nor in Ct.Po (lateral quadrant) between age-/sex-matched donors without diabetes and CKD (*n* = 46; 68.0 ± 20.0 yr) and donors with T2D and CKD or just T2D (*n* = 34; 70.1 ± 13.4 yr) while AGEs were higher in T2D.^50^

The lower cortical porosity in T2D within the femur mid-diaphysis is perhaps surprising (Table 2) given clinical associations between fractures and higher Ct.Po in T2D.^12^ However, lower cortical porosity in T2D subjects compared to non-D subjects has been observed at sites closer to the diaphysis,^51^^,^^52^ and observing higher Ct.Po in T2D can depend on skeletal site (ie, distal tibia vs distal radius)^53^ (Table S1). Thus, T2D-related differences in cortical bone likely depend on the anatomical site being evaluated (porosity varies through the cortex; Figure S1), and whether higher Ct.Po in T2D weakens bone and leads to a fracture can depend on the layer within the cross-section of cortical bone (periosteal vs mid-cortex vs endosteal).^12^

Incubating human cortical bone in ribose to cause an increase AGE content in the organic matrix is known to increase the thermal stability of demineralized bone.^28^^,^^54^ Therefore, the higher temperatures at denaturation of the organic matrix (*T_d_*), onset of heat flow (*T*_onset_), and the peak temperature (*T*_peak_) during heat flow in T2D compared to Ctrl donors (Table 4) may be due to the T2D-related increase in AGEs and/or mature enzymatic collagen crosslinks. The consequence of higher thermal stability of the organic matrix on mechanical properties of cortical bone is difficult to predict. In the present study, the T2D-related increases in *T_d_*, *T*_onset_, and *T*_peak_ did not translate to T2D-related differences in bone strength, toughness, and fracture toughness. In an in vitro study, incubating human cortical bone in ribose, but not glucose, at high concentrations decreased BW.^27^ However, in vivo accumulation of AGEs in the present study did not disrupt the ability of the organic matrix to absorb water via hydrogen bonding.

There are limitations in the present cadaver study that influence the interpretation of the results. We were reliant on the tissue banks (NDRI and MTF) for the status and duration of T2D. Ideally, information on glycated hemoglobin A1c would be available for both donor groups to determine if mechanical properties of human cortical bone are related to glycemic control. History of a fragility fracture was also not provided. Mechanical specimens are commonly extracted from the mid-diaphysis of long bones because of the ease by which uniform test specimens meeting ASTM standards can be machined from this region. We don’t know if systemic changes occurring at sites of osteoporotic fractures like the FN are reflected at the mid-diaphysis, and T2D-related differences in the cortex of the proximal femur remain to be thoroughly investigated. The direction of loading in tension was parallel to the long axis of the osteons, and the crack propagated perpendicular to this osteonal axis during the loading of the SENB. Given the anisotropic material behavior of cortical bone (ie, transversely isotropic), T2D-related differences in tensile properties could exist in radial and circumferential direction. Differences in fracture toughness may exist when the crack propagates along the direction of the osteons. Although we assessed multiple bone properties at different length scales, we did not investigate the complex inter-relationships between different properties, thereby keeping the focus on T2D comparisons with and without standard adjustments. Therefore, it remains to be determined if T2D affects structure-function relationships underlying bone fragility. For example, lower cortical porosity may compensate for the higher PEN or higher fAGEs in predicting mechanical properties of cortical bone. Lastly, the present study did not investigate difference in the fatigue resistance of human cortical bone between T2D and control donors, though it did incorporate non-linear fracture mechanics in the determination of fracture toughness properties. Still, the ability of diabetic bone to resist the accumulation of microdamage may be compromised.

In conclusion, the ultimate tensile stress, post-yield toughness in tension, crack initiation toughness, and crack growth toughness of human cortical bone at the apparent level were not different between cadaveric donors without and with T2D, even though AGEs and thermal stability of the organic matrix were higher in the T2D group. The T2D-related differences in these matrix characteristics likely translated to a higher resistance to tissue-level creep during cyclic micro-indentation (avg-ED) at the femoral mid-diaphysis. Resistance to impact micro-indentation (BMSi) trended to being lower in the T2D than in the control group when accounting for the influence of donor sex, age, and BMI on this tissue-level property. Given that the areal BMD of the proximal femur and cortical thickness of femur diaphysis were higher in T2D compared to age- and sex-matched control donors, the higher-than-expected fracture risk in adults with T2D cannot simply be attributed to deficits in mechanical properties of cortical bone, at least not for bone at the mid-diaphysis. The elevated fracture risk in T2D may be a problem of diabetic comorbidities. Ultimate tensile strength of cortical bone was lower in donors with CKD than donors without CKD when pooling male and female donors with an age range between 50 and 97 yr.

## Supplementary Material

Supplemental_T2D_Differences_in_Bone_Rev3_zjaf173

## Data Availability

Data herein is available upon request.

## References

[1] Fan Y, Wei F, Lang Y, Liu Y. Diabetes mellitus and risk of hip fractures: a meta-analysis. Osteoporos Int. 2016;27(1):219-228. 26264604 10.1007/s00198-015-3279-7

[2] Wang H, Ba Y, Xing Q, Du J-L. Diabetes mellitus and the risk of fractures at specific sites: a meta-analysis. BMJ Open. 2019;9(1):e024067. 10.1136/bmjopen-2018-024067 PMC6326306 30610024

[3] Lipscombe LL, Jamal SA, Booth GL, Hawker GA. The risk of hip fractures in older individuals with diabetes: a population-based study. Diabetes Care. 2007;30(4):835-841. 17392544 10.2337/dc06-1851

[4] Schwartz AV, Vittinghoff E, Bauer DC, et al. Association of BMD and FRAX score with risk of fracture in older adults with type 2 diabetes. JAMA. 2011;305(21):2184-2192. 21632482 10.1001/jama.2011.715 PMC3287389

[5] Eastell R, Vittinghoff E, Lui L, et al. Diabetes mellitus and the benefit of antiresorptive therapy on fracture risk. J Bone Miner Res. 2022;37(11):2121-2131. 36065588 10.1002/jbmr.4697 PMC10092457

[6] Leungsuwan DS, Chandran M. Bone fragility in diabetes and its management: a narrative review. Drugs. 2024;84(9):1111-1134. 39103693 10.1007/s40265-024-02078-5

[7] Bathina S, Armamento-Villareal R. The complex pathophysiology of bone fragility in obesity and type 2 diabetes mellitus: therapeutic targets to promote osteogenesis. Front Endocrinol. 2023;14:1168687. 10.3389/fendo.2023.1168687 PMC10422976 37576965

[8] Hak DJ, Fitzpatrick D, Bishop JA, et al. Delayed union and nonunions: epidemiology, clinical issues, and financial aspects. Injury. 2014;45:S3-S7. 10.1016/j.injury.2014.04.002 24857025

[9] Gao S, Zhao Y. Quality of life in postmenopausal women with osteoporosis: a systematic review and meta-analysis. Qual Life Res. 2023;32(6):1551-1565. 36383282 10.1007/s11136-022-03281-1

[10] Emerzian SR, Johannesdottir F, Yu EW, Bouxsein ML. Use of noninvasive imaging to identify causes of skeletal fragility in adults with diabetes: a review. JBMR Plus. 2024;8(2):ziae003. 38505529 10.1093/jbmrpl/ziae003 PMC10945731

[11] Ferrari S, Akesson KE, Al-Daghri N, et al. Bone microstructure and TBS in diabetes: what have we learned? A narrative review. Osteoporos Int. 2025;36(7):1115-1128. 40353870 10.1007/s00198-025-07495-0

[12] Heilmeier U, Cheng K, Pasco C, et al. Cortical bone laminar analysis reveals increased midcortical and periosteal porosity in type 2 diabetic postmenopausal women with history of fragility fractures compared to fracture-free diabetics. Osteoporos Int. 2016;27(9):2791-2802. 27154435 10.1007/s00198-016-3614-7 PMC6687459

[13] Heilmeier U, Joseph GB, Pasco C, et al. Longitudinal evolution of bone microarchitecture and bone strength in type 2 diabetic postmenopausal women with and without history of fragility fractures—a 5-year follow-up study using high resolution peripheral quantitative computed tomography. Front Endocrinol. 2021;12:599316. 10.3389/fendo.2021.599316 PMC8008748 33796067

[14] Karim L, Moulton J, Vliet MV, et al. Bone microarchitecture, biomechanical properties, and advanced glycation end-products in the proximal femur of adults with type 2 diabetes. Bone. 2018;114:32-39. 29857063 10.1016/j.bone.2018.05.030 PMC6141002

[15] Piccoli A, Cannata F, Strollo R, et al. Sclerostin regulation, microarchitecture, and advanced glycation end-products in the bone of elderly women with type 2 diabetes. J Bone Miner Res. 2020;35(12):2415-2422. 32777114 10.1002/jbmr.4153 PMC8143610

[16] Leanza G, Cannata F, Faraj M, et al. Bone canonical Wnt signaling is downregulated in type 2 diabetes and associates with higher advanced glycation end-products (AGEs) content and reduced bone strength. eLife. 2024;12:RP90437. 38598270 10.7554/eLife.90437 PMC11006415

[17] Hunt HB, Torres AM, Palomino PM, et al. Altered tissue composition, microarchitecture, and mechanical performance in cancellous bone from men with type 2 diabetes mellitus. J Bone Miner Res. 2019;34(7):1191-1206. 30866111 10.1002/jbmr.3711 PMC6650336

[18] Britton M, Monahan GE, Murphy CG, et al. An investigation of composition, morphology, mechanical properties, and microdamage accumulation of human type 2 diabetic bone. Bone. 2024;187:117190. 38960297 10.1016/j.bone.2024.117190

[19] Unal M, Creecy A, Nyman JS. The role of matrix composition in the mechanical behavior of bone. Curr Osteoporos Rep. 2018;16(3):205-215. 29611037 10.1007/s11914-018-0433-0 PMC5948175

[20] Wölfel EM, Fiedler IAK, Kolibova SD, et al. Human tibial cortical bone with high porosity in type 2 diabetes mellitus is accompanied by distinctive bone material properties. Bone. 2022;165:116546. 36113843 10.1016/j.bone.2022.116546

[21] Wölfel EM, Bartsch B, Koldehoff J, et al. When cortical bone matrix properties are indiscernible between elderly men with and without type 2 diabetes, fracture resistance follows suit. JBMR Plus. 2023;7(12):e10839. 38130774 10.1002/jbm4.10839 PMC10731113

[22] McGuigan FE, Malmgren L. Bone health as a co-morbidity of chronic kidney disease. Best Pr Res Clin Rheumatol. 2022; 36(3):101760. 10.1016/j.berh.2022.101760 35718689

[23] Granke M, Makowski AJ, Uppuganti S, Does MD, Nyman JS. Identifying novel clinical surrogates to assess human bone fracture toughness. J Bone Miner Res. 2015;30(7):1290-1300. 25639628 10.1002/jbmr.2452 PMC4478129

[24] Nyman JS, Gorochow LE, Horch RA, et al. Partial removal of pore and loosely bound water by low-energy drying decreases cortical bone toughness in young and old donors. J Mech Behav Biomed. 2013;22:136-145. 10.1016/j.jmbbm.2012.08.013 PMC3655090 23631897

[25] Granke M, Coulmier A, Uppuganti S, Gaddy JA, Does MD, Nyman JS. Insights into reference point indentation involving human cortical bone: sensitivity to tissue anisotropy and mechanical behavior. J Mech Behav Biomed. 2014;37:174-185. 10.1016/j.jmbbm.2014.05.016 PMC4112765 24929851

[26] Uppuganti S, Granke M, Manhard MK, et al. Differences in sensitivity to microstructure between cyclic- and impact-based microindentation of human cortical bone. J Orthop Res. 2016;35(7):1442-1452. 27513922 10.1002/jor.23392 PMC5530367

[27] Nyman JS, Uppuganti S, Unal M, et al. Manipulating the amount and structure of the organic matrix affects the water compartments of human cortical bone. JBMR Plus. 2019;3(6):e10135. 31346566 10.1002/jbm4.10135 PMC6636778

[28] Unal M, Uppuganti S, Dapaah DY, et al. Effect of ribose incubation on physical, chemical, and mechanical properties of human cortical bone. J Mech Behav Biomed Mater. 2023;140:105731. 36827936 10.1016/j.jmbbm.2023.105731 PMC10068591

[29] Willett TL, Dapaah DY, Uppuganti S, Granke M, Nyman JS. Bone collagen network integrity and transverse fracture toughness of human cortical bone. Bone. 2019;120:187-193. 30394355 10.1016/j.bone.2018.10.024 PMC6360115

[30] Iranmanesh F, Dapaah DY, Nyman JS, Willett TL. An improved linear systems model of hydrothermal isometric tension testing to aid in assessing bone collagen quality: effects of ribation and type-2 diabetes. Bone. 2024;186:117139. 38823567 10.1016/j.bone.2024.117139 PMC12103735

[31] Sellmeyer DE, Civitelli R, Hofbauer LC, Khosla S, Lecka-Czernik B, Schwartz AV. Skeletal metabolism, fracture risk, and fracture outcomes in type 1 and type 2 diabetes. Diabetes. 2016;65(7):1757-1766. 27329951 10.2337/db16-0063 PMC4915586

[32] Leslie WD, Lix LM, Prior HJ, Derksen S, Metge C, O’Neil J. Biphasic fracture risk in diabetes: a population-based study. Bone. 2007;40(6):1595-1601. 17392047 10.1016/j.bone.2007.02.021

[33] Oei L, Zillikens MC, Dehghan A, et al. High bone mineral density and fracture risk in type 2 diabetes as skeletal complications of inadequate glucose control. Diabetes Care. 2013;36(6):1619-1628. 23315602 10.2337/dc12-1188 PMC3661786

[34] Vestergaard P . Discrepancies in bone mineral density and fracture risk in patients with type 1 and type 2 diabetes—a meta-analysis. Osteoporosis Int. 2007;18(4):427-444. 10.1007/s00198-006-0253-4 17068657

[35] Vestergaard P, Rejnmark L, Mosekilde L. Diabetes and its complications and their relationship with risk of fractures in type 1 and 2 diabetes. Calcified Tissue Int. 2009;84(1):45. 10.1007/s00223-008-9195-5 19067021

[36] Melton LJ, Leibson CL, Achenbach SJ, Therneau TM, Khosla S. Fracture risk in type 2 diabetes: update of a population-based Study1^*^. J Bone Miner Res. 2008;23(8):1334-1342. 18348689 10.1359/JBMR.080323 PMC2574704

[37] Cirovic A, Vujacic M, Petrovic B, et al. Vascular complications in individuals with type 2 diabetes mellitus additionally increase the risk of femoral neck fractures due to deteriorated trabecular microarchitecture. Calcif Tissue Int. 2022;110(1):65-73. 34302494 10.1007/s00223-021-00894-5 PMC8302969

[38] Cirovic A, Schmidt FN, Vujacic M, et al. Lower microhardness along with less heterogeneous mineralization in the femoral neck of individuals with type 2 diabetes mellitus indicates higher fracture risk. JBMR Plus. 2024;8(3):ziae005. 38741606 10.1093/jbmrpl/ziae005 PMC11090112

[39] Arjunan D, Rastogi A, Ghosh J, et al. Trabecular and cortical bone microarchitecture using high-resolution peripheral quantitative computed tomographic imaging in diabetic peripheral neuropathy. Diabetes Metab Syndr. 2024;18(8):103109. 39191163 10.1016/j.dsx.2024.103109

[40] Oren TW, Botolin S, Williams A, Bucknell A, King KB. Arthroplasty in veterans: analysis of cartilage, bone, serum, and synovial fluid reveals differences and similarities in osteoarthritis with and without comorbid diabetes. J Rehabilitation Res Dev. 2011;48(10):1195. 10.1682/jrrd.2010.09.0186 PMC4487361 22234664

[41] Sihota P, Yadav RN, Dhaliwal R, et al. Investigation of mechanical, material, and compositional determinants of human trabecular bone quality in type 2 diabetes. J Clin Endocrinol Metab. 2021;106(5):e2271-e2289. 33475711 10.1210/clinem/dgab027

[42] Yang J, Zhang Y, Liu X, Chen B, Lei L. Effect of type 2 diabetes on biochemical markers of bone metabolism: a meta-analysis. Front Physiol. 2024;15:1330171. 39100278 10.3389/fphys.2024.1330171 PMC11294215

[43] Sihota P, Kumar S, Dhaliwal R, et al. Multi-scale inferomedial femoral neck bone quality in type 2 diabetes patients with fragility fracture. Bone. 2025;192:117375. 39694129 10.1016/j.bone.2024.117375

[44] Rufus-Membere P, Holloway-Kew KL, Diez-Perez A, Kotowicz MA, Pasco JA. Associations between bone impact microindentation and clinical risk factors for fracture. Endocrinology. 2019;160(9):2143-2150. 31310275 10.1210/en.2019-00415

[45] Samakkarnthai P, Sfeir JG, Atkinson EJ, et al. Determinants of bone material strength and cortical porosity in patients with type 2 diabetes mellitus. J Clin Endocrinol Metab. 2020;105(10):e3718-e3729. 32556277 10.1210/clinem/dgaa388 PMC7458544

[46] Rufus-Membere P, Holloway-Kew KL, Diez-Perez A, et al. Reference intervals for bone impact microindentation in healthy adults: a multi-centre international study. Calcif Tissue Int. 2023;112(3):338-349. 36729139 10.1007/s00223-022-01047-y PMC9968254

[47] Sundh D, Rudäng R, Zoulakis M, Nilsson AG, Darelid A, Lorentzon M. A high amount of local adipose tissue is associated with high cortical porosity and low bone material strength in older women. J Bone Miner Res. 2015;31(4):749-757. 26588353 10.1002/jbmr.2747

[48] Rufus-Membere P, Holloway-Kew KL, Diez-Perez A, Kotowicz MA, Pasco JA. Associations between bone material strength index, calcaneal quantitative ultrasound and bone mineral density in men. J Endocr Soc. 2020;5(4):bvaa179. 33728389 10.1210/jendso/bvaa179 PMC7940167

[49] Emerzian SR, Chow J, Behzad R, et al. Long-duration type 1 diabetes is associated with deficient cortical bone mechanical behavior and altered matrix composition in human femoral bone. J Bone Miner Res. 2024;40(1):87-99. 39561104 10.1093/jbmr/zjae184 PMC11700620

[50] Dapaah DY, Arakawa S, Carroll GA, et al. Advanced glycation end-product adducts alter the bone collagen network and human cortical bone fracture resistance. JBMR Plus. 2025;ziaf172. 41503150 10.1093/jbmrpl/ziaf172 PMC12771375

[51] Nilsson AG, Sundh D, Johansson L, et al. Type 2 diabetes mellitus is associated with better bone microarchitecture but lower bone material strength and poorer physical function in elderly women: a population-based study. J Bone Miner Res. 2017;32(5):1062-1071. 27943408 10.1002/jbmr.3057

[52] Agarwal S, Germosen C, Rosillo I, et al. Fractures in women with type 2 diabetes are associated with marked deficits in cortical parameters and trabecular plates. J Bone Miner Res. 2024;39(8):1083-1093. 38861455 10.1093/jbmr/zjae091 PMC11337576

[53] Samelson EJ, Demissie S, Cupples LA, et al. Diabetes and deficits in cortical bone density, microarchitecture, and bone size: Framingham HR-pQCT study. J Bone Miner Res. 2017;33(1):54-62. 28929525 10.1002/jbmr.3240 PMC5771832

[54] Willett TL, Sutty S, Gaspar A, Avery N, Grynpas M. In vitro non-enzymatic ribation reduces post-yield strain accommodation in cortical bone. Bone. 2013;52(2):611-622. 23178516 10.1016/j.bone.2012.11.014

