# Supplementary material for: Differences and similarities in cortical bone of the femur between donors with and without type 2 diabetes
Source: J Bone Miner Res. 2025 Nov 21;41(4):434–46. doi: 10.1093/jbmr/zjaf173 (PMC13034673; doi:10.1093/jbmr/zjaf173)
Supplement: Supplemental_T2D_Differences_in_Bone_Rev3_zjaf173 [file supplemental_t2d_differences_in_bone_rev3_zjaf173.docx]

**Differences and Similarities in Cortical Bone of the Femur between Donors with and without Type 2 Diabetes**

Emily Berestesky, Sasidhar Uppuganti, Daniel Y. Dapaah, Daniel Fernandes, Kara Donovan, Nick Livingston, David Lutsky, Mong Zhang, Alicia M. Hymel, Jacquelyn Pennings, Paul Voziyan, Mark D. Does, Thomas L. Willett, and Jeffry S. Nyman

**Table of Contents**

Table S1. Abbreviations and Acronyms. . . . . . . . . . . . . . . . . . . . . . . . . . . . . . . . . . . . . . . . . . S-2

Table S2. Differences and similarities in bone between subjects with and without T2D using non-invasive and minimally invasive techniques. . . . . . . . . . . . . . . . . . . . . . . . . . . . . . . . . . . . . . . . . . . . . . . . . . S-3

Table S3. Estimated coefficients (β) and p-values from linear regression models for selected bone properties in the T2D group analysis . . . . . . . . . . . . . . . . . . . . . . . . . . . . . . . . . . . . . . . . . . . . . . . . . . . . . . S-10

Table S4. Estimated coefficients (β) and p-values from linear regression models predicting fracture toughness properties based on μCT properties of the specimens, group, sex, and age. . . . . . . . . . . . . . S-12

Table S5. Differences in selected bone properties between no CKD and CKD. . . . . . . . . . . S-13

Table S6. Estimated coefficients (β) and p-values from linear regression models for selected bone properties in the CKD group analysis. . . . . . . . . . . . . . . . . . . . . . . . . . . . . . . . . . . . . . . . . . . . . . . . . . . . . . S-15

Figure S1: Figure S1: Representative grayscale, segmented, and three-dimensional images and renderings from μCT scans of each cadaveric mid-diaphysis of the femur. . . . . . . . . . . . . . . . . . . . . . . . S-16

Figure S2: Representative force vs. displacement curve from cyclic reference point indentation. . . . . . . . . . . . . . . . . . . . . . . . . . . . . . . . . . . . . . . . . . . . . . . . . . . . . . . . . . . . . . . . . . . . . . . . . . . . . . . . . . . . . . S-17

Figure S3: Linear regression of avg-CID of the femur mid-diaphysis vs. age . . . . . . . . . . . . S-17

Figure S4: Linear regression of Bone Material Strength Index (BMSi) of the femur mid-diaphysis vs. Age or BMI. . . . . . . . . . . . . . . . . . . . . . . . . . . . . . . . . . . . . . . . . . . . . . . . . . . . . . . . . . . . . . . . . . . . . . S-18

References cited in Supplemental Materials. . . . . . . . . . . . . . . . . . . . . . . . . . . . . . . . . . . . . . S-18

**Table S1. Abbreviations and Acronyms**.

| **aBMD**, Areal bone mineral density  **ADA**, American Diabetes Association  **ANCOVA**, Analysis of covariance  **avg-CID**, Average creep indentation distance  **avg-ED**, Average energy dissipated  **avg-LS**, Average loading slope  **avg-US**, Average unloading slope  **BMI**, Body mass index  **BMSi**, Bone material strength index  **BV/TV**, Trabecular bone volume fraction  **CKD**, Chronic kidney disease  **cRPI**, Cyclic reference point indentation  **CSA**, Cross-sectional area  **Ct.Ar**, Cortical bone area  **Ct.avBMD**, Average cortical volumetric bone mineral density  **Ct.Po**, Cortical porosity  **Ct.Po.Dm**, Mean cortical pore diameter  **Ct.Po.V**, Cortical pore volume  **Ct.Th**, Cortical thickness  **Ct.TMD**, Cortical tissue mineral density  **Ct.vBMD**, Cortical volumetric bone mineral density  **Ctrl**, Control donors without diabetes  **DCE-**MRI, Dynamic contrast-enhanced magnetic resonance imaging  **ΔH**, enthalpy of denaturation  **Dm.SD**, Distribution of cortical pore diameters  **DPD**, Deoxypyridinoline  **DR**, Distal radius  **DSC**, Differential Scanning Calorimetry  **DT**, Distal tibia  **DXA**, Dual-energy x-ray absorptiometry  **EDTA**, ethlenediaminetatraacetic acid  **fAGEs**, Fluorescent advanced glycation end-products  **FBG**, Fasting blood glucose  **FN**, Femoral neck  **FRAX**, Fracture Risk Assessment Tool  **FWHM**, Full width at half maximum (enthalpy)  **H NMR**, Proton nuclear magnetic resonance  **HA**, Hemi-arthroplasty  **HbA1c**, Hemoglobin A1c blood test  **HIT**, Hydrothermal isometric tension  **HP**, hydroxylysyl-pyridinoline  **HPLC**, high-performance liquid chromatography  **HR-pQCT**, high resolution, peripheral quantitative computer tomography  **Hyp**, Hydroxyproline  **IDI**, Indentation distance increase  **IFG**, Impaired fasting glucose  **IGT**, Impaired glucose tolerance | **IMI**, Impact micro-indentation  **I_min_/c_min_**, Section modulus  **Jf**, Energy dissipated during crack growth until failure  **K_grow_**, Rate of crack propagation  **K_init_**, Critical stress intensity for crack initiation  **M**, Men  **MRI**, Magnetic resonance imaging  **MVD**, Microvascular disease  **NDRI**, National Disease Research Interchange  **NGM**, Normal glucose metabolism  **Non-D**, Non-diabetic control subjects  **OA**, Osteoarthritis  **OGTT**, Oral glucose tolerance test  **PEN**, Pentosidine  **PG**, Plasma glucose  **PMW**, Post-menopausal women  **PN**, Peripheral neuropathy  **PRD**, Proximal radial diaphysis  **PTD**, Proximal tibial diaphysis  **PY strain**, Post-yield strain at 0.2%  **PYD**, Pyridinoline  **SD**, Standard Deviation  **T2D**, Type 2 diabetes  **Tb.Sp**, Trabecular spacing  **Tb.Sp.SD**, Standard deviation of intertrabecular distances  **Tb.Th**, Trabecular thickness  **Tb.vBMD**, Trabecular volumetric bone mineral density  **Tb.N**, Trabecular number  **TcPO_2_**, Transcutaneous oxygen tension  **T_d_**, Denature temperature  **THA**, Total hip arthroplasty  **TID**, Total indentation distance  **T_onset_** , Temperature at onset of heat flow  **Tot.BMD**, Total bone mineral density  **Tot.vBMD**, Total volumetric bone mineral density  **T_peak_** , Maximum recorded temperature during heat flow  **Tt.Ar**, Total cross-sectional area  **UR**, Ultradistal radius  **UT**, Ultradistal tibia  **vBMD**, Volumetric bone mineral density  **W**, Women  **WHO**, World Health Organization  **μCT**, microcomputed tomography |
| --- | --- |

**Table S2. Differences and similarities in bone between subjects with and without T2D using non-invasive and minimally invasive techniques**.

| **Study**  **Design ^a^** | **Modality ^b^** | **Sex (years) ^c^** | **Groups (sample size) ^d^** | **T2D Duration**  **(years) ^e^** | **T2D-related finding ^f^** | **T2D effect** | **Ref** |
| --- | --- | --- | --- | --- | --- | --- | --- |
| CS | HR-pQCT of DR and DT | PMW | Non-D (19) vs. T2D (19) | ≥ 5 | Higher vBMD, due to greater Tb.vBMD and Tb.Th (DT), and Higher Ct.Po.V and Ct.Po (DR). | ↑ in bone mass | ^(1)^ |
| CS | HR-pQCT of UR, DR, UT, and DT | PMW | Non-D/Ctrl (20) vs. Non-D/Fx (20) vs. T2D/Ctrl (20) vs. T2D/Fx (20) | ≥ 3 | Greater Ct.Po (DR, UT, & DT) in T2D/Fx vs. T2D. Lower Ct.vBMD in T2D/Fx vs. T2D group. No differences between Ctrl and Fx w/in Non-D. | ↑ in cortical porosity when fracture occurs in T2D but not in Non-D | ^(2)^ |
| CS | HR-pQCT of DR and DT | PMW | Non-D (14) vs. T2D (14); T2D defined as FPG >126mg/dl | 1 – 30 | No differences except lower Ct.Ar (DT). | ↔ in bone mass; ↓ in bone area | ^(3)^ |
| CS | MRI of DR | PMW | Non-D (21) vs. T2D (14) | ≥ 5 | Baseline-CS: No differences in trabecular architecture between groups. | ↔ in bone architecture | ^(4)^ |
| L |  |  |  |  | L: Average follow-up time was 1.9 yrs - No differences in follow-up changes in trabecular architecture when adjusted for ethnicity *. | ↔ changes in bone architecture |  |
| CS | OsteoProbe of tibia | PMW | Non-D (30) vs. T2D (30) | ≥ 10 | Lower BMSi in T2D than in non-D without or with adjusting for BMI *. | ↓ in bone matrix quality | ^(5)^ |
|  | HR-pQCT of DR and DT |  |  |  | Higher Ct.Th (DR & DT), Tb.N (DR), and BV/TV (DT) without adjustment; No differences after adjusting for BMI *. | BMI-related ↑ in bone architecture |  |
| CS | HR-pQCT of DR and DT | W (59.6 ± 2.6) | Non-D (78) vs. T2D FBG ≥ 126 mg/dl (22) | 1 to ≥ 12 | After adjustment for smoking, osteoporosis medication, glucocorticoid and thiazolinedione use, greater Ct.Po (DR) *. No differences between groups (DT). | ↑ in cortical porosity at non-weight bearing site | ^(6)^ |
| CS | Axial QCT of proximal femur | PMW | Non-D/Ctrl (19) vs. Non-D/Fx (19) vs. T2D/Ctrl (20) vs. T2D/Fx (19) | ≥ 3 | Lower femoral neck (FN) vBMD and lower cortical thickness in T2D/Fx compared to T2D/Ctrl. No difference between Ctrl and Fx within Non-D. Smaller minimum cross-sectional area of FN in T2D compared to Non-D, regardless of Fx status. | ↓ bone mass with a fracture in T2D but not in Non-D | ^(7)^ |
| CS | OsteoProbe of tibia | PMW | Non-D (19) vs. T2D (16); T2D defined as HbA1c ≥ 6.5% | 14.3 ± 2.0 | 9.2% lower BMSi. Inverse relationship between BMSi and T2D duration. | ↓ in bone matrix quality | ^(8)^ |
| CS | HR-pQCT of DR and DT | W (76.4 ± 2.6) & M (76.1 ± 2.5) | Non-D (144 W, 159 M) vs. T2D (11 W,18 M); T2D defined by antidiabetic treatment | Not reported | After adjusting for weight *, no differences between T2D and Non-D M and W (DR and DT). | ↔ in bone mass; ↔ in bone architecture | ^(9)^ |
| CS | HR-pQCT of DR and DT | PMW | Non-D/Ctrl (19-20) vs. Non-D/Fx (20) vs. T2D/Ctrl (17-19) vs. T2D/Fx (18-20); T2D as classified by ADA & on antidiabetic treatment for ≥ 3 years | T2D: 7.6 ± 3.1  T2D/Fx: 13.3 ± 8.8 | Greater Ct.Po (DR and DT) in T2D/Fx compared to T2D/Ctrl. *Differences were more pronounced at the DR with higher porosity in the mid-cortical and periosteal layer, respectively, of T2D/Fx vs. T2D/Ctrl* **^#^**. No differences in porosity between non-diabetic groups at either skeletal site. | ↑ in cortical porosity when fracture occurs in T2D but not in Non-D | ^(10)^ |
| CS | HR-pQCT of DR and DT | W & M  (T2D.MVD-: 51.4 ± 11.1, Co.MVD-: 51.3 ±11.3, T2D.MVD+: 65.3 ± 7.1, Co.MVD+: 65.1 ± 6.7) | Non-D w/o Microvascular disease (26) vs. Non-D w/ MVD (25) vs. T2D w/o MVD (26) vs. T2D w/ MVD (25). T2D defined by WHO criteria and medication use | T2D w/o MVD: 10.0 ± 5.6  T2D w/ MVD: 17.2 ± 7.1 | After adjusting for BMI *, lower Ct.vBMD (DR) in T2D with MVD than in T2D w/o MVD. No differences between non-MVD and MVD within Non-D. | ↓ in T2D cortical bone mass with co-occurrence of MVD | ^(11)^ |
| CS | HR-pQCT of DR and DT | W & M  (40 – 87) | Non-D (552 W, 388 M) vs. T2D (54 W, 75 M) | 5 – 34 | *Lower Ct.BMD and higher Ct.Po at DT but no such differences at DR* **^#^** when adjusted for age, sex, weight and height as well as when stratified by prior fracture; No differences in estimated failure load. | ↓ in cortical bone mass; ↑ in cortical porosity at weight-bearing site | ^(12)^ |
| CS | OsteoProbe of tibia | PMW | Non-D (954) vs. T2D (99); T2D defined as medication use or FPG > 7 mmol/L or RPG > 11 mmol/L | 2 – 25 | Lower BMSi after adjustment for 7 covariates *. | ↓ in bone matrix quality | ^(13)^ |
|  | HR-pQCT of DR and DT as well as proximal radial and tibial diaphysis (PRD, PTD) |  |  |  | Higher BV/TV and Tb.N (DR and DT); Lower Tb.Sp (DR only); Higher total BMD and Ct.Ar (DR and PRD); *Higher Ct.Po (DR) but lower Ct.Po (PRD)* **^#^**; Higher Ct.vBMD (PRD and PTD) and higher estimated failure load (PRD, PTD and DR) after adjustment for covariates *. | ↑ in bone mass; ↑ in predicted bone strength |  |
| CS | HR-pQCT of UR and UT | W (Non-D: 55 ± 8.1, T2D: 58 ± 14.29) & M (Non-D: 57 ± 11.19, T2D: 57 ± 11.11) | Non-D (16 W, 26 M) vs. T2D (17 W, 26 M); T2D defined by medication use & HbA1c 6 – 10% | W: 8.21 ± 5.82  M: 7.71 ± 7.12 | Greater Tb.N (UR and UT) and greater Ct.Th (UR). | ↑ in trabecular architecture and cortical structure | ^(14)^ |
| CS | HR-pQCT of DR and DT | W & M (Non-D: 56.8 ± 8.3, PDM: 59.9 ± 8.0, T2D: 62.5 ± 7.1) | Non-D (152 W, 127 M) vs. Prediabetes (PDM) (34 W, 32 M) vs. T2D (24 W, 41 M); Based on OGTT, Non-D, those w/ NGM; PDM, those w/ IFG or IGT; T2D, those w/ FPG ≥ 126.0 mg/dL & 2-h PG ≥ 200.0 mg/dL | 0 – 8 | After adjusting for 6  covariates *, PDM associated with lower Tb.N (DT) and T2D associated with smaller CSA (DT). HbA1c > 7% associated with lower Ct.BMD and Ct.Th, higher Tb.N and Ct.Po (DR), and higher Tb.N and lower Tb.Th (DT). T2D duration > 5 years associated with higher Tb.N (DR). | Poor glycemic control negatively associated with cortical bone mass and thickness | ^(15)^ |
| CS | HR-pQCT of DR, PRD, DT, and PTD | PMW | Non-D (50) vs. T2D (42). T2D as defined by ADA | 11.2 ± 7.6 | After adjusting for years post-menopause, race and weight, higher trabecular BV/TV, Tb.vBMD (DT and DR), Tb.Th and estimated stiffness (DR); Higher BV/TV and Tb.vBMD (DR and DT) more pronounced for patients with < 10 years duration of T2D compared to ≥ 10 years. Elevated estimated stiffness (PRD and PTD) and lower cortical load fraction (PRD) in T2D < 10 years but not in ≥ 10 years. | ↑ in bone mass but this may diminish with disease progression | ^(16)^ |
| CS | OsteoProbe of tibia | Black & white PMW & M  (≥ 50) | Non-D (50 W,6 B) vs. Prediabetes (PDM) (75 W, 13 B) vs. Early stage T2D (24 W, 16 B); PDM defined as FPG < 100 mg/dl & HbA1c ≤ 5.6%; PDM defined as FPG 100 – 125 mg/dL and/or HbAc15.7-6.4%; T2D defined by ADA | ≤ 5 | After adjusting for age, sex, BMI and urine pentosidine levels, lower BMSi for black T2D subjects compared to controls and PDM, and BMSi was lower for T2D black subjects compared to T2D white subjects (no differences amongst white groups). | ↓ in bone matrix quality during low duration T2D for African Americans but not for Caucasians | ^(17)^ |
| CS | OsteoProbe of tibia | PMW & M (Non-D: 67.3 ± 8.8, T2D: 68.8 ± 7.6) | Non-D (63 M & 45 M) vs. T2D (75 W & 96 M) | 15.4 ± 6.4 | No significant differences in BMSi but higher skin AGEs in T2D when adjusted for age, sex, and BMI; Negative correlation between BMSi and skin AGEs in T2D and in non-T2D. | ↓ in bone matrix quality as AGEs ↑ | ^(18)^ |
|  | HR-pQCT of DR and DT |  |  |  | Higher Ct.Ar and estimated failure load (DR) when adjusted for age, sex, and BMI; Negative correlation between Ct.Po (DR and DT) and transcutaneous oxygen tension (TcPO_2_) in T2D patients with peripheral vascular disease (TcPO_2_ < 40 mm Hg). | ↑ in cortical porosity as peripheral vascular disease worsens |  |
| CS  & L | HR-pQCT of UT and UR | PMW | Non-D (12) vs. T2D (10) vs. T2D/Fx (10) | T2D: 6.4 ± 4.2 years  T2D/Fx: 11.2 ± 8.0 years | Baseline-CS: Highest Ct.Po in T2D/Fx vs. other groups (UT).  L: After 4-5 years, all groups had increased Ct.Po at similar annual rates (UT); T2D/Fx had greatest annual decrease in failure load and stiffness vs. Non-D and T2D. | Similar rate increase in porosity and ↓ in bone strength in T2D/Fx | ^(19)^ |
| CS | OsteoProbe of tibia | M (54.0 – 73.6) | Non-D (234) vs. IFG (59) vs. T2D (47); IFG defined as fasting plasma glucose (FPG) of 5.5 - 6.9 mmol/L; T2D defined as FPG ≥ 7.0 mmol/L & by ADA | 5.7 – 18.6 | After adjusting for 5  covariates *, lower BMSi compared to those with impaired fasting glucose (IFG) and Non-D. | ↓ in bone matrix quality | ^(20)^ |
| CS | HR-pQCT of DR and DT | W & M  (Non-D: 57.7 ± 15.8, T1D: 52.9 ± 15.3, T2D: 62.1 ± 10.1) | Non-D (160) vs. T1D (109) vs. T2D (96); T1D & T2D defined as HbA1c ≥ 6.5% | T1D: 26.44 ± 14.2  T2D: 11.4 ± 9.3 | After adjusting for 14 covariates*, higher Tt.BMD, Ct.BMD, Tb.BMD, Ct.Ar, and Ct.Th (DT and DR); Higher Tb.N and Lower Tb.Sp (DR) but lower Tb.Sp in DT. | ↑ in bone mass | ^(21)^ |
| CS | HR-pQCT of DR, DT, and PTD | PMW | Non-D (390) vs. T2D (175); T2D determined by physician or HbA1c ≥ 6.5%. T2D/Fx (59) vs. T2D/Ctrl (103) | Not clarified | After adjusting for 8  covariates *, higher Tb.vBMD, Tb.N, Ct.Ar, lower Tb.S and heterogeneity (DR); *higher* Tb.vBMD,Tb.N, Ct.Ar, Ct.Th, *Ct.Po, Ct.Po.Dm* and lower Ct.vBMD at DT but higher Ct.vBMD *and lower Ct.Po at PTD* **^#^** without Fx. Lower Tot.vBMD, Ct.Ar, Ct.Th and Ct.vBMD and estimated failure load (DR) in T2D/Fx compared to T2D. Lower Ct.Ar, Ct.Th, Ct.vBMD and estimated failure load (DT and PTD) in T2D/Fx compared to T2D group. | ↑ in bone mass regardless of fracture status and ↓ in trabecular structure and bone strength with fracture | ^(22)^ |
| CS | HR-pQCT of DR, DT, and PTD | M (Non-D: 84.47 ± 4.25, T2D: (84.29 ± 4.01) | Non-D (1,487) vs. T2D (290); T2D self-reported or medication use | 16.22 ± 14.07 | After adjusting for 6 covariates*, lower Tt.Ar and Ct.Ar (DT and PTD), Ct.vBMD and estimated failure load (PTD); T2D duration ≥ 10 years was associated with greater Tb.Th (DR and DT) and *with Ct.Po at DR but not at DT and PTD* **^#^**; men with HbAc1 ≥ 7.5% correlated with greater Ct.Ar, Ct.Th, and estimated failure load (PTD). | ↓ in bone structure at distal tibia and proximal tibia diaphysis only  ↑ in cortical porosity with T2D duration at distal radius only  ↑ in trabecular thickness with increasing T2D duration | ^(23)^ |
| CS | HR-pQCT of DR and DT | W & M  (52 – 64) | T2D w/o peripheral neuropathy (PN) (T2D) (98) vs. W/ PN (T2D/PN) (198); T2D self-reported and medication usage and PN define by absence of 10-g monofilament perception & vibration perception threshold < 25 msec on either foot | T2D: 6 – 13.3  T2D/PN: 8 – 20 | PN subjects had increased Ct.Po (DT) and Tb.N (DT and DR); After adjusting for 9 covariates, PN correlated with decreased Tot.vBMD, Ct.vBMD and increased Ct.Po (DT); Negative correlations between Tot.vBMD and Ct.vBMD with duration of T2D and Tot.vBMD with HbAc1 (DT); Positive correlation between Ct.Po and duration of T2D. | ↓ in bone mass with peripheral diabetic neuropathy | ^(24)^ |
| CS | HR-pQCT of UT | PMW & M (61.8 ± 5.4) | Non-D w/o MVD (28) vs. Non-D w/ MVD (9) vs T2D w/ MVD (21) vs. T2D w/o MVD (17); T2D > 3 years, as defined by ADA. MVD defined as ≥ 1 presence of neuropathy, nephropathy, or retinopathy | > 3  11.3 ± 7.6 | After adjusting for age, sex, and BMI, increased Ct.Po.Dm and Ct.Po.Dm.SD (DT); Increased Ct.Po and Ct.Po.V and decreased Ct.vBMD (DT) in MVD vs. non-MVD when adjusted for T2D status. | ↑ cortical porosity especially with co-occurrence of micro-vascular disease | ^(25)^ |
|  | DCE-MRI of UT |  |  |  | After adjusting for age, sex, and BMI, increased average vessel volume and vessel diameter in T2D. After further adjusting for T2D status, increased mean vessel density, and decreased mean vessel volume and diameter in MVD subjects. | ↑ vasculature but ↓ circulation load |  |
| CS | HR-pQCT of DT and DR | Obese M (MHO: 47.1 ± 9.3, MUHO w/o T2D: 50.1 ± 7.1, MUHO w/ T2D: 53.7 ± 7.9) | Metabolically healthy obese (MHO) (15) vs. Metabolically unhealthy obese (MUHO) w/o T2D (56) vs. MUHO with T2D (41). T2D defined with antidiabetic medications, HbA1C ≥ 6.5%, or FBG ≥ 126 mg/dL | 0.25 – 19 | After adjusting for age, free testosterone, and free estradiol, men with T2D had greater Tb.Sp, lower estimated failure load, and lower estimated stiffness (DT) and greater Tb.Sp (DR). | ↓ in trabecular architecture and ↓ bone strength | ^(26)^ |
| CS | HR-pQCT of DT and DR | PMW (64.2 *±* 6.7) | T2D with no sarcopenia (17) vs. T2D with probable sarcopenia (77) vs. T2D with non-obese sarcopenia (18) vs. T2D with obese sarcopenia (18). T2D defined as HbA1c > 7-10% | 10 (7 – 12) | Lower Tot/Ct.vBMD, BV/TV, Tb.N, Tb.Th, and Ct.Th (DR and DT) in sarcopenia than in no or probable sarcopenia. *Higher Ct.Po with sarcopenia at DT but not DR* **^#^**. | ↓ in trabecular architecture with sarcopenia, regardless if T2D is obese | ^(27)^ |

^a^ Cross-sectional (CS) or Longitudinal (L) study

^b^ distal tibia (DT); proximal tibial diaphysis (PTD); proximal radial diaphysis (PRD); distal radius (DR); ultra-distal radius (UR); ultra-distal tibia (UT); proximal radius (PR); Non-diabetic (Non-D) w/o fragility fracture (Ctrl) or w/ fragility fracture (Fx)

^c^ Participant age information provided when available: mean ± standard deviation or minimum – maximum range for men and women, Post-menopausal women (PMW), Women (W), and Men (M)

^d^ No diabetes (Non-D), type 2 diabetes (T2D), fracture (Fx), micro-vascular disease (MVD), Prediabetes (PDM)

^e^ Duration in years given as greater than, the state range, or mean ± SD

^f^ See Table S2 for abbreviations

* Adjusted for covariates, which are listed if for four or less factors.

**^#^** T2D-related differences in cortical porosity depends on skeletal site.

**Table S3. Estimated coefficients (β) and p-values from linear regression models for selected bone properties in the T2D group analysis.**

| **Property** | **Units** | **Reference** | **Group (T2D)** | **Sex (Female)** | **Age (yr)** | **BMI (kg/m^2^)** | **Adj-R^2^** |
| --- | --- | --- | --- | --- | --- | --- | --- |
| Hip aBMD | (g/cm^2^) | β_0_ = +1.16  p<0.001 | β_1_ = +0.05  **p=0.046** | β_2_ = -0.15  **p<0.001** | β_3_ = -0.0045  **p<0.001** | β_4_ = +0.0016,  p=0.276 | 0.321 |
| Hip T-score ^a^ |  | β_0_ = +2.13  p=0.017 | β_1_ = +0.37  *p=0.070* | β_2_ = -1.27  **p<0.001** | β_3_ = -0.037  **p<0.001** | β_4_ = +0.018,  p=0.129 | 0.350 |
| FN T-score ^a^ |  | β_0_ = +2.43  p=0.010 | β_1_ = +0.36,  p=0.098 | β_2_ = -1.08  **p<0.001** | β_3_ = -0.047  **p<0.001** | β_4_ = +0.015,  p=0.224 | 0.326 |
| Ct.Ar | (mm^2^) | β_0_ = +5.28,  p<0.001 | β_1_ = +0.35,  **p=0.004** | β_2_ = -1.37  **p<0.001** | β_3_ = -0.0129  **p=0.011** | β_4_ = +0.0200,  **p=0.004** | 0.589 |
| Ct.Th | (mm) | β_0_ = +8.26,  p<0.001 | β_1_ = +0.70,  **p=0.004** | β_2_ = -1.36  **p<0.001** | β_3_ = -0.0255  **p=0.011** | β_4_ = +0.0241,  p=0.080 | 0.321 |
| Ct.Po | (%) | β_0_ = -6.80,  p=0.121 | β_1_ = -2.86,  **p=0.008** | β_2_ = +1.20  p=0.243 | β_3_ = +0.18  **p<0.0001** | β_4_ = +0.06,  p=0.202 | 0.147 |
| Avg-CID | (μm) | β_0_ = +1.45,  p<0.001 | β_1_ = -0.12,  **p= 0.005** | β_2_ = +0.15,  **p<0.001** | β_3_ = -0.0027,  p=0.129 | β_4_ = +0.0018,  p=0.475 | 0.166 |
| Avg-ED | (μJ) | β_0_ = +53.5,  p<0.001 | β_1_ = -0.7,  p=0.579 | β_2_ = +4.7,  **p<0.001** | β_3_ = -0.1279,  **p=0.019** | β_4_ = +0.0260,  p=0.727 | 0.156 |
| BMSi | (mm/mm) | β_0_ = +77.6,  p<0.001 | β_1_ = -1.9,  *p=0.096* | β_2_ = -5.7,  **p<0.001** | β_3_ = +0.108,  **p=0.026** | β_4_ = +0.159,  **p=0.018** | 0.230 |
| T_d_ | (°C) | β_0_ = +63.7,  p<0.001 | β_1_ = +0.6,  **p=0.005** | β_2_ = +0.3,  p=0.194 | β_3_ = -0.0072,  p=0.417 | β_4_ = +0.0027,  p=0.822 | 0.066 |
| T_peak_ | (°C) | β_0_ = +59.9,  p<0.001 | β_1_ = +0.86,  **p=0.001** | β_2_ = +0.53,  **p=0.041** | β_3_ = -0.0131,  p=0.243 | β_4_ = +0.0178,  p=0.250 | 0.146 |
| T_onset_ | (°C) | β_0_ = +53.5,  p<0.001 | β_1_ = +0.75,  **p=0.014** | β_2_ = +1.07,  **p<0.001** | β_3_ = -0.03,  **p=0.015** | β_4_ = +0.05,  **p=0.009** | 0.286 |
| PEN | (mmol/mol col) | β_0_ = -0.059,  p=0.789 | β1 = +0.27,  **p<0.001** | β_2_ = -0.05,  p=0.324 | β_3_ = +0.0109,  **p<0.001** | β_4_ = +0.0026,  p=0.382 | 0.332 |
| PYD | (mmol/mol col) | β_0_ = +212.5,  p<0.001 | β_1_ = +20.6,  **p=0.010** | β_2_ = +14.4,  *p=0.060* | β_3_ = -0.8516,  **p=0.011** | β_4_ =0.2930,  p=0.522 | 0.146 |
| DPD | (mmol/mol col) | β_0_ = +128.9,  p<0.001 | β_1_ = +12.4,  **p=0.029** | β_2_ = +16.3,  **p=0.003** | β_3_ = -0.5217,  **p=0.028** | β_4_ = +0.2919,  p=0.371 | 0.166 |
| fAGE | (ng quinine/mol col) | β_0_ = +49.28,  p<0.001 | β_1_ = +6.63,  **p=0.002** | β_2_ = +0.85,  p=0.680 | β_3_ = +0.0173,  p=0.848 | β_4_ = -0.0125  p= 0.920 | 0.053 |
| *μCT of Tensile Beams* | |  |  |  |  |  |  |
| Ct.Po | (%) | β_0_ = +3.938,  p=0.283 | β_1_ = -0.918,  p=0.279 | β_2_ = +0.470,  p=0.566 | β_3_ = +0.0290,  p=0.418 | β_4_ = -0.0332,  p=0.501 | -0.002 |
| Ct.TMD | (mgHA/cm^3^) | β_0_ = +1072,  p<0.001 | β_1_ =+2.0,  p=0.594 | β_2_ = -9.6,  **p=0.008** | β_3_ = +0.0668,  p=0.667 | β_4_ = -0.1502,  p=0.484 | 0.043 |
| Ct.vBMD | (mgHA/cm^3^) | β_0_ =1032,  p<0.001 | β_1_ = +10.8,  p=0.251 | β_2_ = -13.6,  p=0.135 | β_3_ = -0.1917,  p=0.627 | β_4_ = +0.1536,  p=0.778 | 0.002 |
| *μCT of SENB* |  |  |  |  |  |  |  |
| Ct.Po | (%) | β_0_ = +3.059,  p=0.436 | β_1_ = -1.890,  **p=0.039** | β_2_ =+0.475,  p=0.589 | β_3_ = +0.0300,  p=0.435 | β_4_ = -0.0420,  p= 0.428 | 0.033 |
| Ct.TMD | (mgHA/cm^3^) | β_0_ = +1004,  p<0.001 | β_1_ = +4.3,  p=0.195 | β_2_ = -10.2,  **p=0.002** | β_3_ = +0.0925,  p=0.512 | β_4_ = -0.2557,  p=0.190 | 0.091 |
| Ct.vBMD | (mgHA/cm^3^) | β_0_ = +977.9,  p<0.001 | β_1_ = +20.8,  **p=0.025** | β_2_ = -14.1,  p=0.113 | β_3_ = -0.1731,  p=0.654 | β_4_ = 0.1150,  p=0.829 | 0.039 |

^a^ Hip and FN T-scores are estimates since cadaveric bone samples were scanned without soft tissue.

Significant values are bolded; trending values are italicized in blue.

**Table S4.** **Estimated coefficients (β) and p-values from linear regression models predicting fracture toughness properties based on μCT properties of the specimens, group, sex, and age.**

| **Model** | **Reference** | **Ct.Po (%)**  **Ct.vBMD (mgHA/cm^3^)** | **Group (T2D)** | **Sex (Female)** | **Age (yr)** |
| --- | --- | --- | --- | --- | --- |
| K_init_ = Ct.Po + Group + Age + Sex | β_0_ = +7.74  p<0.0001 | β_1_ = -0.07  **p=0.021** | β_1_ = -0.19  p=0.498 | β_2_ = +0.60  **p=0.036** | β_3_ = -0.008  p=0.465 |
| J_f_ = Ct.Po + Group + Age + Sex | β_0_ = +12.77  p<0.0001 | β_1_ = -0.13  **p=0.025** | β_1_ = -0.70  p=0.203 | β_2_ = +1.24  **p=0.024** | β_3_ = -0.06  **p=0.008** |
| K_grow_ = Ct.Po + Group + Age + Sex | β_0_ = +11.22  p<0.0001 | β_1_ = -0.14  **p=0.021** | β_1_ = +0.07  p=0.902 | β_2_ = -1.13  *p=0.053* | β_3_ = -0.05  *p=0.054* |
| K_init_ = Ct.vBMD + Group + Age + Sex | β_0_ = +2.05  p=0.508 | β_1_ = +0.006  *p=0.059* | β_1_ = -0.17  p=0.553 | β_2_ = +0.64  **p=0.027** | β_3_ = -0.01  p=0.384 |
| J_f_ = Ct.vBMD + Group + Age + Sex | β_0_ = +6.80  p=0.259 | β_1_ = +0.006  p=0.310 | β_1_ = -0.55  p=0.323 | β_2_ = +1.26  **p=0.024** | β_3_ = -0.06  **p=0.005** |
| K_grow_ = Ct.vBMD + Group + Age + Sex | β_0_ = +1.23  p=0.847 | β_1_ = +0.01  p=0.109 | β_1_ = +0.16  p=0.902 | β_2_ = -1.06  *p=0.075* | β_3_ = -0.05  **p=0.038** |

**Table S5. Differences in selected bone properties between no CKD and CKD**.

| **Characteristic** | **(Units)** | **No CKD ^a^** | **CKD ^a^** | **p-value ^b^** | ***p-value ^c^** |
| --- | --- | --- | --- | --- | --- |
| *DXA of proximal femur* ^d^ | | n=86 | n=30 |  |  |
| Hip aBMD | (g/cm^2^) | 0.839 (0.731, 0.974) | 0.811 (0.646, 0.946) | 0.222 | **0.003** |
| FN aBMD | (g/cm^2^) | 0.724 (0.615, 0.829) | 0.686 (0.549, 0.788) | 0.180 | **0.003** |
| *μCT of femur diaphysis* | | n=88 | n=31 |  |  |
| I_min_/c_min_ | (cm^3^) | 1.56 (1.19, 1.98) | 1.82 (1.29, 2.03) | 0.439 | **0.015** |
| Ct.Ar | (cm^2^) | 3.88 (3.29, 4.74) | 4.36 (3.08, 5.00) | 0.476 | **0.028** |
| Tt.Ar | (cm^2^) | 6.05 (5.31, 6.90) | 6.34 (5.54, 7.28) | 0.255 | *0.061* |
| Ct.Th | (mm) | 5.99 (4.98, 7.05) | 6.33 (4.74, 7.37) | 0.603 | 0.338 |
| Ct.TMD | (mgHA/cm^3^) | 861 (848, 870) | 863 (848, 872) | 0.624 | 0.144 |
| Ct.avBMD | (mgHA/cm^3^) | 835 (810, 859) | 835 (810, 853) | 0.639 | 0.666 |
| Ct.Po | (%) | 5.78 (2.96, 9.22) | 7.93 (3.54, 10.4) | 0.205 | 0.662 |
| *cRPI of periosteal surface* | | n=88 | n=31 |  |  |
| TID | (mm) | 81.3 (77.5, 88.3) | 81.4 (77.8, 84.3) | 0.368 | 0.415 |
| IDI | (mm) | 12.0 (11.2, 13.4) | 11.6 (10.4, 13.1) | 0.217 | 0.481 |
| avg-CID | (mm) | 1.45 (1.32, 1.59) | 1.35 (1.23, 1.53) | *0.096* | 0.343 |
| avg-ED | (mJ) | 46.4 (42.0, 52.8) | 47.8 (40.8, 52.2) | 0.748 | 0.232 |
| avg-US | (N/mm) | 0.420 (0.384,0.442) | 0.418 (0.376, 0.458) | 0.855 | 0.810 |
| avg-LS | (N/mm) | 0.562 (0.510, 0.604) | 0.581 (0.517, 0.603) | 0.325 | 0.291 |
| *IMI of periosteal surface* | | n=88 | n=31 |  |  |
| BMSi |  | 88.4 (83.3, 93.2) | 91.0 (86.5, 93.4) | 0.172 | 0.638 |
| *Tensile test of cortical bone* | | n=88 | n=31 |  |  |
| Yield Stress | (MPa) | 96.0 (89.4, 103.2) | 91.8 (86, 101.4) | *0.074* | *0.066* |
| Ultimate Stress | (MPa) | 108.7 (99.9, 116.0) | 104.6 (95.7, 111.3) | **0.035** | *0.055* |
| Yield Strain | (%) | 0.77 (0.76, 0.79) | 0.77 (0.75, 0.78) | 0.108 | 0.425 |
| Failure Strain | (%) | 1.97 (1.58, 2.41) | 1.97 (1.51, 2.48) | 0.677 | 0.838 |
| PY Strain | (%) | 1.19 (0.80, 1.62) | 1.21 (0.76, 1.72) | 0.818 | 0.891 |
| Toughness | (MJ/m^3^) | 1.69 (1.19, 2.24) | 1.50 (1.14, 1.96) | 0.365 | 0.497 |
| PY toughness | (MJ/m^3^) | 1.22 (0.77, 1.73) | 1.07 (0.73, 1.56) | 0.495 | 0.662 |
| *Fracture toughness test of cortical bone* | | n=88 | n=31 |  |  |
| K_init_ | MPa·m^0.5^ | 7.26 (6.31, 8.18) | 6.56 (5.54, 7.71) | *0.068* | 0.416 |
| J_f_ | kJ/m^2^ | 8.0 (6.7, 9.9) | 7.3 (6.4, 10.2) | 0.212 | 0.367 |
| K_grow_ | MPa·m^0.5^/mm | 5.78 (4.49, 7.60) | 6.19 (5.23, 8.01) | 0.280 | 0.930 |
| *HIT test of demineralized cortical bone* | | n=88 | n=31 |  |  |
| Max Slope | (kPa) | 46.9 (42.5, 51.8) | 46.6 (39.4, 53.4) | 0.827 | 0.876 |
| T_d_ | (°C) | 63.1 (62.4, 63.9) | 63.2 (62.7, 64.1) | 0.314 | 0.332 |
| *DSC of demineralized cortical bone* | | n=88 | n=31 |  |  |
| T_onset_ | (°C) | 52.5 (51.5, 54.1) | 52.8 (52.4, 54.2) | 0.760 | 0.643 |
| T_peak_ | (°C) | 59.2 (58.2, 60.2) | 59.7 (58.6, 60.1) | 0.243 | 0.173 |
| ΔH | (°C) | 9.9 (8.6, 10.9) | 9.3 (8.1, 10.4) | 0.139 | 0.427 |
| *HPLC and fluorescence* | | n=88 | n=30-31 |  |  |
| PEN | (mmol / mol col) | 0.574 (0.407, 0.739) | 0.692 (0.588, 0.917) | **0.006** | 0.151 |
| PYD | (mmol / mol col) | 153 (128, 191) | 169 (114, 190) | 0.938 | 0.901 |
| DPD | (mmol / mol col) | 100 (81, 124) | 110 (58, 130) | 0.609 | 0.359 |
| fAGE | (ng quinine / mol col) | 44.9 (39.1, 55.2) | 47.6 (38.5, 54.8) | 0.655 | 0.657 |
| *^1^H NMR of cortical bone* | | n=88 | n=31 |  |  |
| Bound water | (%) | 20.2 (19.4, 20.7) | 20.1 (19.5, 20.7) | 0.683 | 0.240 |
| Pore water | (%) | 5.25 (4.5, 6.7) | 5.5 (4.5, 6.7) | 0.570 | 0.196 |

^a^ Median (interquartile range)

^b^ Wilcoxon rank sum test (i.e., Mann-Whitney U test)

^c^ *p-value from an analysis of covariance in which sex (male or female) and group (No CKD or CKD) were categorical variables while age (years) and BMI (kg/m^2^) were continuous variables.

**Table S6. Estimated coefficients (β) and p-values from linear regression models for selected bone properties in the CKD group analysis.**

| **Property** | **Units** | **Reference** | **Group (CKD)** | **Sex (Female)** | **Age (yr)** | **BMI (kg/m^2^)** | **Adj-R^2^** |
| --- | --- | --- | --- | --- | --- | --- | --- |
| Hip aBMD | (g/cm^2^) | β_0_ = +1.13,  p<0.001 | β_1_ = -0.087,  **p=0.003** | β_2_ = -0.17,  **p<0.001** | β_3_ = -0.0039,  **p<0.001** | β_4_ = +0.0034,  **p=0.022** | 0.350 |
| FN aBMD | (g/cm^2^) | β_0_ = +1.02,  p<0.001 | β_1_ = -0.080,  **p=0.003** | β_2_ = -0.13,  **p<0.001** | β_3_ = -0.0045,  **p<0.001** | β_4_ = +0.0033,  **p=0.014** | 0.359 |
| I_min_/c_min_ | (cm^3^) | β_0_ = +1.87,  p<0.001 | β_1_ = -0.17,  **p=0.015** | β_2_ = -0.76,  **p<0.001** | β_3_ = -0.0028,  p=0.279 | β_4_ = +0.0136,  **p<0.001** | 0.592 |
| Ct.Ar | (cm^2^) | β_0_ = +4.73,  p<0.001 | β_1_ = -0.31,  **p=0.028** | β_2_ = -1.43,  **p<0.001** | β_3_ = -0.0103,  **p=0.046** | β_4_ = +0.0293  **p<0.001** | 0.577 |
| Tt.Ar | (cm^2^) | β_0_ = +5.94,  p<0.001 | β_1_ = -0.31,  *p=0.061* | β_2_ = -1.67,  **p<0.001** | β_3_ = +0.0048,  p=0.433 | β_4_ = +0.0268  **p=0.002** | 0.550 |
| Ultimate stress | (MPa) | β_0_ = +123.3,  p<0.001 | β_1_ = -5.0,  *p=0.055* | β_2_ = +1.176,  p=0.598 | β_3_ = -0.2416  **p=0.012** | β_4_ = +0.0671,  p=0.602 | 0.083 |
| Yield Stress | (MPa) | β_0_ = +1.0,  p<0.001 | β_1_ = -0.04,  p=0.425 | β_2_ = -0.05,  p=0.287 | β_3_ = -0.0015,  p=0.463 | β_4_ = -0.0009,  p=0.750 | -0.017 |
| K_init_ | MPa·m^0.5^ | β_0_ = +9.14,  p<0.001 | β_1_ = -0.27,  p=0.416 | β_2_ = +0.51,  *p=0.083* | β_3_ = -0.0183,  p=0.145 | β_4_ = -0.0271,  p=0.111 | 0.046 |
| PEN | (mmol / mol col) | β_0_ = -0.399,  p=0.091 | β_1_ = +0.09370,  p=0.151 | β_2_ = -0.03492,  p=0.536 | β_3_ = +0.0117,  **p<0.001** | β_4_ = +0.0062,  *p=0.058* | 0.185 |


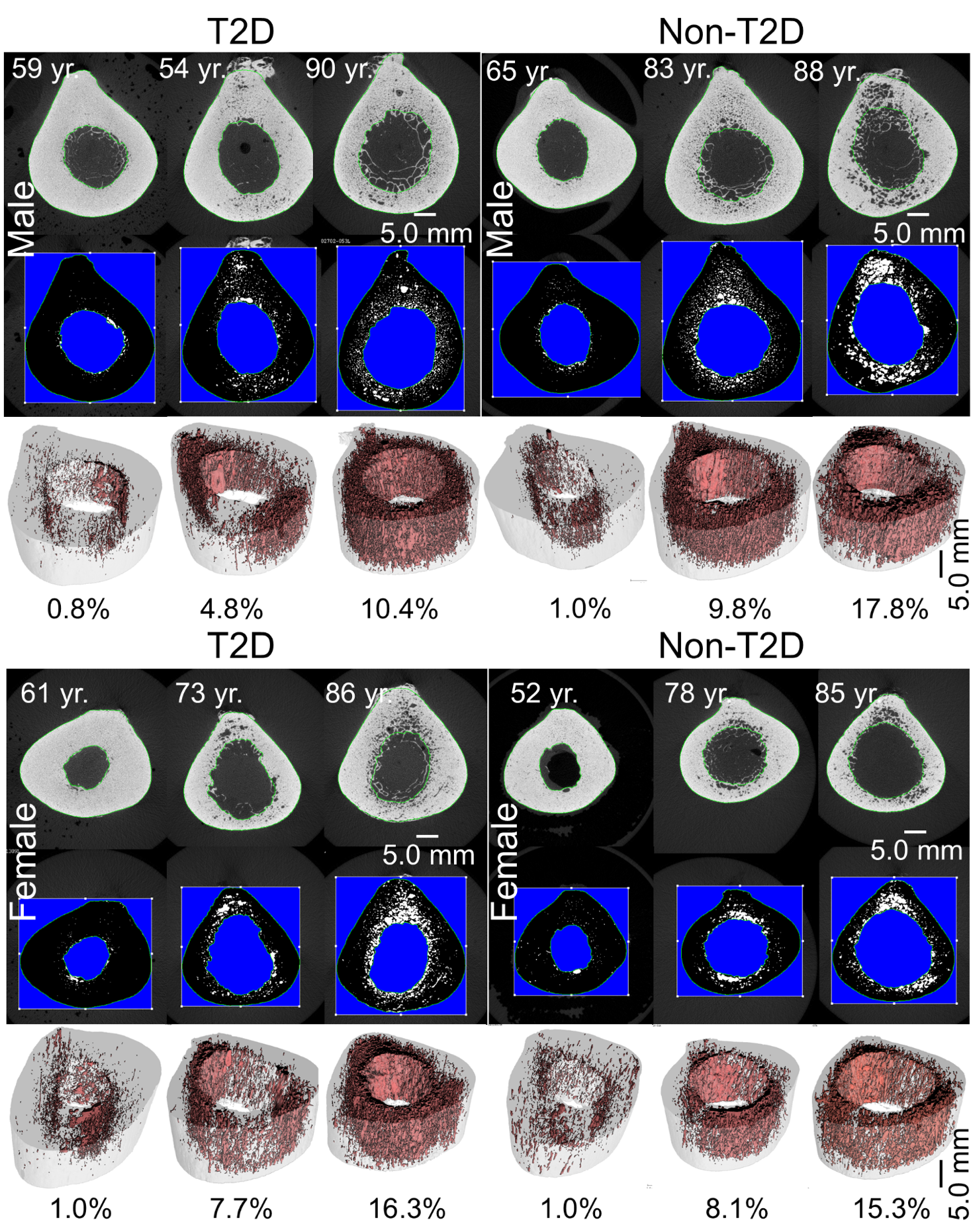


**Figure S1: Representative grayscale, segmented, and three-dimensional images and renderings from μCT scans of each cadaveric mid-diaphysis of the femur.** The intra-cortical porosity (%) of the femur mid-diaphysis was determined with an inverted threshold range (-500 <mgHA/cm^3^< 441.5) and a Gaussian noise filter (sigma=0.2, support=1.0) to segment the pore or void spaces from bone. Contours were fit to the periosteal and endosteal surfaces to define the region of interest as the cortex, thereby skipping trabecular-like bone that was present for certain donors.


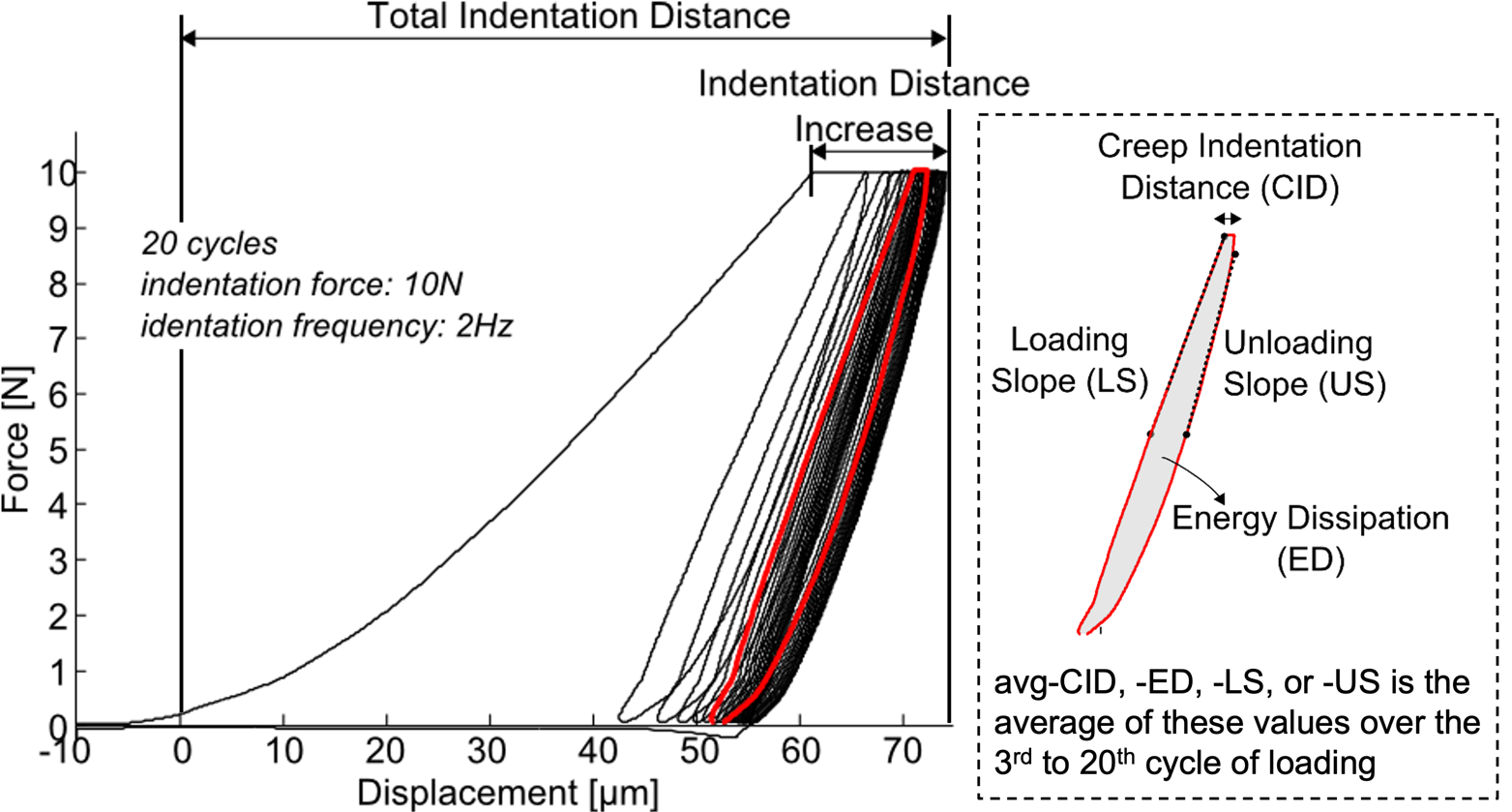


**Figure S2: Representative force vs. displacement curve from cyclic reference point indentation**. The resistance of the cortical bone to micro-indentation is characterized by multiple properties as depicted in the graph.


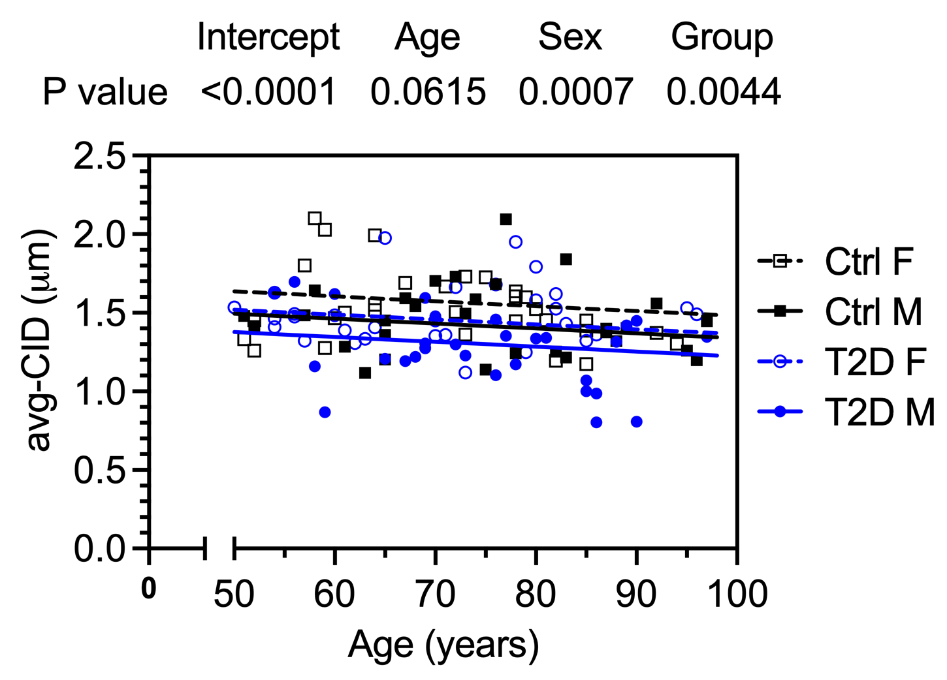


**Figure S3: Linear regression of avg-CID of the femur mid-diaphysis vs. age**. At a given age, creep indentation distance (CID) averaged (avg-) over the 3^rd^ to last cycle of loading was low for donors who had T2D than for donor who didn’t have diabetes. The age-related decrease in avg-CID was modest (shallow slope). P-values come from ANCOVA in which Group (Ctrl or T2D) and Sex (Male, M, or Female, F) are categorical variables. For the estimates of coefficients for the full model that includes BMI, which was not a significant explanatory variable, see Table S2.


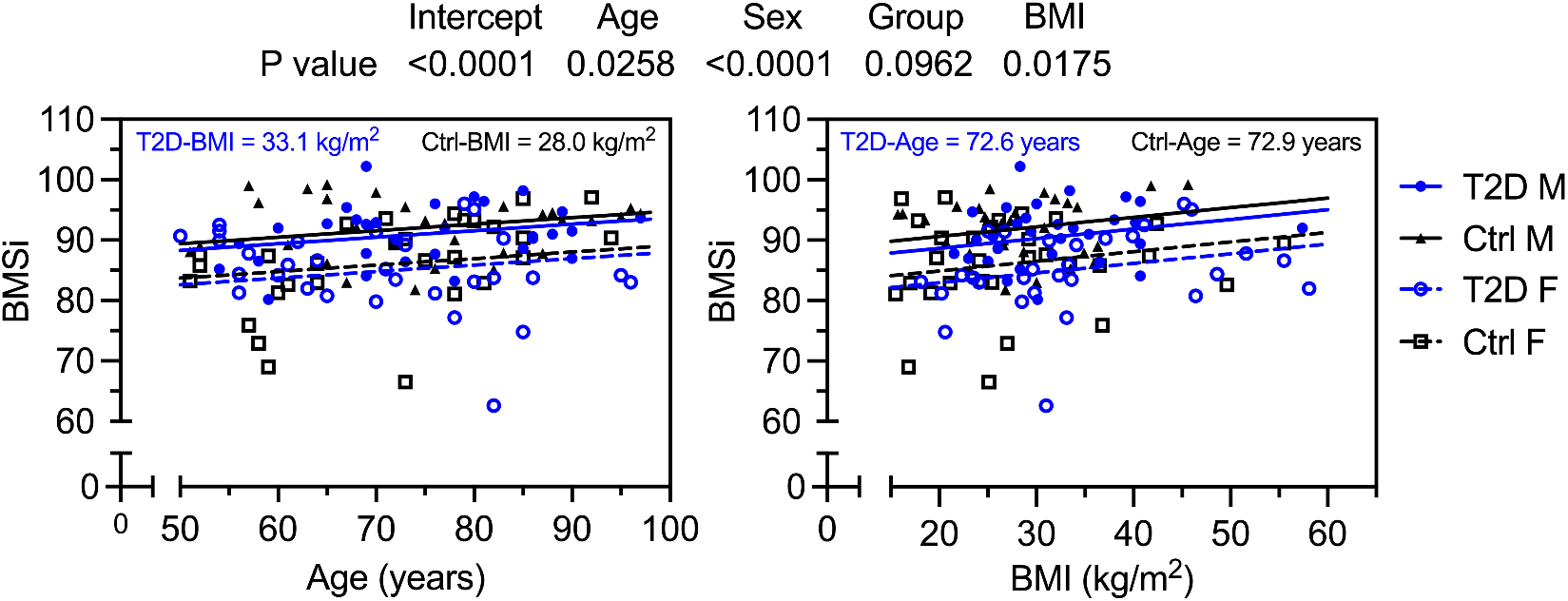


**Figure S4: Linear regression of Bone Material Strength Index (BMSi) of the femur mid-diaphysis vs. Age or BMI**. Bone material strength index depended on both age and body mass index such that BMSi was higher for male donors than female donors at a given age or BMI. Although the effect of T2D on BMSi was not strictly significant (0.05 < p-value < 0.1), this resistance to micro-indentation property tended to be lower in T2D than in Ctrl for a given age or BMI. The mean BMI per diabetes group and mean Age per diabetes group were used to generate the different regression lines. P-values come from general linear regressions in which Group (Ctrl or T2D) and Sex (Male, M, or Female, F) are categorical variables. For the estimates of coefficients, see Table S3.

**References cited in Supplemental Materials**

1. Burghardt AJ, Issever AS, Schwartz AV, Davis KA, Masharani U, Majumdar S, Link TM. High-Resolution Peripheral Quantitative Computed Tomographic Imaging of Cortical and Trabecular Bone Microarchitecture in Patients with Type 2 Diabetes Mellitus. J. Clin. Endocrinol. Metab. 2010 Nov;95(11):5045–55.

2. Patsch JM, Burghardt AJ, Yap SP, Baum T, Schwartz AV, Joseph GB, Link TM. Increased cortical porosity in type 2 diabetic postmenopausal women with fragility fractures. J. Bone Miner. Res. 2013 Feb;28(2):313–24.

3. Shu A, Yin MT, Stein E, Cremers S, Dworakowski E, Ives R, Rubin MR. Bone structure and turnover in type 2 diabetes mellitus. Osteoporos. Int. 2012 Feb;23(2):635–41.

4. Pritchard JM, Giangregorio LM, Atkinson SA, Beattie KA, Inglis D, Ioannidis G, Gerstein H, Punthakee Z, Adachi JD, Papaioannou A. Changes in trabecular bone microarchitecture in postmenopausal women with and without type 2 diabetes: a two year longitudinal study. BMC Musculoskelet. Disord. 2013;14(1):114.

5. Farr JN, Drake MT, Amin S, Melton LJ, McCready LK, Khosla S. In Vivo Assessment of Bone Quality in Postmenopausal Women With Type 2 Diabetes. J Bone Miner Res. 2014 Apr;29(4):787–95.

6. Yu EW, Putman MS, Derrico N, Abrishamanian-Garcia G, Finkelstein JS, Bouxsein ML. Defects in cortical microarchitecture among African-American women with type 2 diabetes. Osteoporos. Int. 2014 Nov 15;26(2):673–9.

7. Heilmeier U, Carpenter DR, Patsch JM, Harnish R, Joseph GB, Burghardt AJ, Baum T, Schwartz AV, Lang TF, Link TM. Volumetric femoral BMD, bone geometry, and serum sclerostin levels differ between type 2 diabetic postmenopausal women with and without fragility fractures. Osteoporos. Int. 2015 Apr;26(4):1283–93.

8. Furst JR, Bandeira LC, Fan W-W, Agarwal S, Nishiyama KK, McMahon DJ, Dworakowski E, Jiang H, Silverberg SJ, Rubin MR. Advanced Glycation Endproducts and Bone Material Strength in Type 2 Diabetes. J. Clin. Endocrinol. Metab. 2016 June;101(6):2502–10.

9. Paccou J, Ward KA, Jameson KA, Dennison EM, Cooper C, Edwards MH. Bone Microarchitecture in Men and Women with Diabetes: The Importance of Cortical Porosity. Calcif. Tissue Int. 2016 May;98(5):465–73.

10. Heilmeier U, Cheng K, Pasco C, Parrish R, Nirody J, Patsch JM, Zhang CA, Joseph GB, Burghardt AJ, Schwartz AV, Link TM, Kazakia G. Cortical bone laminar analysis reveals increased midcortical and periosteal porosity in type 2 diabetic postmenopausal women with history of fragility fractures compared to fracture-free diabetics. Osteoporos. Int. 2016 Sept;27(9):2791–802.

11. Shanbhogue VV, Hansen S, Frost M, Jørgensen NR, Hermann AP, Henriksen JE, Brixen K. Compromised cortical bone compartment in type 2 diabetes mellitus patients with microvascular disease. Eur. J. Endocrinol. 2016 Feb;174(2):115–24.

12. Samelson EJ, Demissie S, Cupples LA, Zhang X, Xu H, Liu C, Boyd SK, McLean RR, Broe KE, Kiel DP, Bouxsein ML. Diabetes and Deficits in Cortical Bone Density, Microarchitecture, and Bone Size: Framingham HR‐pQCT Study. J. Bone Miner. Res. 2017 Sept 20;33(1):54–62.

13. Nilsson AG, Sundh D, Johansson L, Nilsson M, Mellström D, Rudäng R, Zoulakis M, Wallander M, Darelid A, Lorentzon M. Type 2 Diabetes Mellitus Is Associated With Better Bone Microarchitecture But Lower Bone Material Strength and Poorer Physical Function in Elderly Women: A Population‐Based Study. J. Bone Miner. Res. 2017 Jan;32(5):1062–71.

14. Patsch JM, Rasul S, Huber FA, Leitner K, Thomas A, Kocijan R, Boutroy S, Weber M, Resch H, Kainberger F, Schüller-Weidekamm C, Kautzky-Willer A. Similarities in trabecular hypertrophy with site-specific differences in cortical morphology between men and women with type 2 diabetes mellitus. PLoS ONE. 2017 Apr;12(4):e0174664.

15. Waard EAC de, Jong JJA de, Koster A, Savelberg HHCM, Geel TA van, Houben AJHM, Schram MT, Dagnelie PC, Kallen CJ van der, Sep SJS, Stehouwer CDA, Schaper NC, Berendschot TTJM, Schouten JSAG, Geusens PPMM, Bergh JPW van den. The association between diabetes status, HbA1c, diabetes duration, microvascular disease, and bone quality of the distal radius and tibia as measured with high-resolution peripheral quantitative computed tomography—The Maastricht Study. Osteoporos. Int. 2018 Dec;29(12):2725–38.

16. Starr JF, Bandeira LC, Agarwal S, Shah AM, Nishiyama KK, Hu Y, McMahon DJ, Guo XE, Silverberg SJ, Rubin MR. Robust Trabecular Microstructure in Type 2 Diabetes Revealed by Individual Trabecula Segmentation Analysis of HR‐pQCT Images. J. Bone Miner. Res. 2018 May 11;33(9):1665–75.

17. Dawson-Hughes B, Bouxsein M, Shea K. Bone material strength in normoglycemic and hyperglycemic black and white older adults. Osteoporos. Int. 2019 Dec;30(12):2429–35.

18. Samakkarnthai P, Sfeir JG, Atkinson EJ, Achenbach SJ, Wennberg PW, Dyck PJ, Tweed AJ, Volkman TL, Amin S, Farr JN, Vella A, Drake MT, Khosla S. Determinants of Bone Material Strength and Cortical Porosity in Patients with Type 2 Diabetes Mellitus. J. Clin. Endocrinol. Metab. 2020 Oct 1;105(10):e3718–29.

19. Heilmeier U, Joseph GB, Pasco C, Dinh N, Torabi S, Darakananda K, Youm J, Carballido-Gamio J, Burghardt AJ, Link TM, Kazakia GJ. Longitudinal Evolution of Bone Microarchitecture and Bone Strength in Type 2 Diabetic Postmenopausal Women With and Without History of Fragility Fractures—A 5-Year Follow-Up Study Using High Resolution Peripheral Quantitative Computed Tomography. Front. Endocrinol. 2021;12:599316.

20. Holloway-Kew KL, Betson A, Rufus-Membere PG, Gaston J, Diez-Perez A, Kotowicz MA, Pasco JA. Impact microindentation in men with impaired fasting glucose and type 2 diabetes. Bone. 2021;142:115685.

21. Rasmussen NH, Dal J, Kvist AV, Bergh JP van den, Jensen MH, Vestergaard P. Bone parameters in T1D and T2D assessed by DXA and HR-pQCT – A cross-sectional study: The DIAFALL study. Bone. 2023;172:116753.

22. Agarwal S, Germosen C, Rosillo I, Bucovsky M, Colon I, Kil N, Wang Z, Dinescu A, Guo X-DE, Walker M. Fractures in women with type 2 diabetes are associated with marked deficits in cortical parameters and trabecular plates. J. Bone Miner. Res. 2024;39(8):1083–93.

23. Faraj M, Schwartz AV, Burghardt AJ, Black D, Orwoll E, Strotmeyer ES, Vittinghoff E, Fogolari M, Angeletti S, Banfi G, Lombardi G, Woods G, Lui L-Y, Bouxsein M, Napoli N. Risk Factors for Bone Microarchitecture Impairments in Older Men With Type 2 Diabetes—The MrOS Study. J. Clin. Endocrinol. Metab. 2024;dgae452.

24. Arjunan D, Rastogi A, Ghosh J, Mukherjee S, Singh R, Dhiman V, Bhadada SK. Trabecular and cortical bone microarchitecture using high-resolution peripheral quantitative computed tomographic imaging in diabetic peripheral neuropathy. Diabetes Metab. Syndr.: Clin. Res. Rev. 2024;18(8):103109.

25. Löffler MT, Wu P, Pirmoazen AM, Joseph GB, Stewart JM, Saeed I, Liu J, Schafer AL, Schwartz AV, Link TM, Kazakia GJ. Microvascular disease not type 2 diabetes is associated with increased cortical porosity: A study of cortical bone microstructure and intracortical vessel characteristics. Bone Rep. 2024;20:101745.

26. Vigevano F, Gregori G, Colleluori G, Chen R, Autemrongsawat V, Napoli N, Qualls C, Villareal DT, Armamento-Villareal R. In Men With Obesity, T2DM Is Associated With Poor Trabecular Microarchitecture and Bone Strength and Low Bone Turnover. J. Clin. Endocrinol. Metab. 2021;106(5):1362–76.

27. Pal R, Prasad TN, Bhadada SK, Singla V, Yadav U, Chawla N. Association between bone microarchitecture and sarcopenia in postmenopausal women with type 2 diabetes. Arch. Osteoporos. 2024;19(1):94.
